# Entropic-dielectric interplay governs ion adsorption in inner electric double layers

**DOI:** 10.1126/sciadv.aee9469

**Published:** 2026-05-15

**Authors:** Matteo Olgiati, Florian Altmann, Moritz Zelenka, Joanna Dziadkowiec, Andreas Kretschmer, Alper T. Celebi, Laura L. E. Mears, Ellen H. G. Backus, Markus Valtiner

**Affiliations:** ^1^ Vienna University of Technology, Institute of Applied Physics, Wiedner Hauptstrasse 8-10, A-1040 Vienna, Austria.; ^2^ University of Vienna, Faculty of Chemistry, Institute of Physical Chemistry, Währinger Straße 42, 1090 Vienna, Austria.; ^3^ University of Vienna, Vienna Doctoral School in Chemistry (DoSChem), Währinger Str. 42, 1090 Vienna, Austria.; ^4^ NJORD Centre, Department of Physics, University of Oslo, Oslo 0371, Norway.

## Abstract

Ion-specific effects at aqueous interfaces are fundamental to chemistry, biology, and environmental science, yet unraveling their thermodynamic origins remains challenging. Here, we quantitatively resolve interfacial thermodynamics at molecular resolution by combining concentration-dependent adsorption isotherms obtained from atomic force microscopy imaging, molecular dynamics simulations, and sum-frequency generation spectroscopy. We show that strongly hydrated ions disrupt and compact interfacial water structure, leading to entropic penalties and increased dielectric screening that weakens their interaction with the surface. Conversely, weakly hydrated ions incur lower entropy costs and substantially decrease screening via water depletion, promoting inner-sphere overadsorption on a charged surface. This balance between entropic penalties and screening governs inner double-layer formation. Our findings establish a thermodynamically grounded molecular framework for ion-specific adsorption, quantitatively linking interfacial entropy, water structure, and electrostatics. This approach generalizes the Hofmeister concept and provides a foundational approach for understanding interfacial structure in complex electrolytes.

## Teaser

Quantitative imaging and simulation reveal how entropy shapes ion-specific double-layer structure.

## INTRODUCTION

Interfacial solvent and ion dynamics play a central role in diverse fields including energy storage (*1*), electrochemistry (*2*), tribology (*3*), biological systems (*4*, *5*), and contact electrification (*6*, *7*). For example, the solvent structure steers electron transfer processes by modulating the electronic couplings between reactants and products, with far-reaching implications, including the inverse Marcus effect (*8*) and ion-specific moderation of electrocatalytic reaction rates (*9*, *10*). Further, adsorbed ions aid ultralow friction in hydration lubricated aqueous tribosystems (*11*, *12*) and control adhesive interactions in aqueous systems (*13*). Despite widespread importance, a detailed atomistic understanding of solvated solid-liquid interfaces remains limited due to their dynamic complexity and the multicomponent nature of the interactions that must be probed, essentially with single-molecular layer resolution. Interfacial thermodynamics and structural dynamics—essential aspects for predicting interfacial physics—are still not easily accessible through experiments or theory alone, a challenge most clearly revealed by an inability to evaluate interfacial thermodynamics and characterize ion adsorption. An integrated experimental and theoretical framework is therefore required to capture complex solvent-solute-surface regulated dynamic structures at solid-liquid interfaces.

A centrally accepted interpretation underlying ion adsorption is ion-specific binding to (charged) surface groups, while quantitative thermodynamic origins for this complex solvent-solute-surface dependent process remain elusive in literature (*14*). Ion-specific effects, which govern hydration shell structure via ion-dipole interactions, exhibit complex behavior that only partly follows frameworks like the Hofmeister series (*15*).

While initial guidance can be drawn from historic kosmotropic/chaotropic (Hofmeister) classifications, hydrophobic ion character, or qualitative analyses of hydrogen-bond networks (*16*–*18*), such concepts are generally regarded as qualitative descriptors of ion-specific trends rather than rigorously defined thermodynamic observables. Therefore, these frameworks inevitably oversimplify the intricate interplay of enthalpic and entropic contributions governing ion-specific effects. Interfacial ion adsorption patterns can be steered by water-induced correlations between the ions (*19*–*22*), whereas, in the bulk, long-ranged water-structure perturbations are still largely debated (*23*–*25*). Capturing interfacial behavior thus demands models that go beyond mean-field approaches, accounting for finite ion and solvent sizes, specific adsorption, and the interplay of ion-solvent, lateral ion-ion, and ion-surface interactions.

We combine experimental and theoretical investigation together with quantitative thermodynamic modeling at solid-liquid interfaces. This methodological combination proves to be essential for identifying the origin of the governing forces as a balance between electrostatic (ion-surface, ion-water, and ion-ion) and entropic contributions, mediated by interfacial dielectric variations. Specifically, using quantitative atomic force microscopy (AFM) imaging of ion adsorption on muscovite mica, molecular dynamics (MD) simulations, and sum-frequency generation (SFG) spectroscopy, we quantify how ion-specific structuring of interfacial water induces entropic penalties, alters dielectric properties, and modifies interfacial polarizability, all of which contribute to a quantitative thermodynamic description.

## RESULTS

### Interfacial ion structure

**Fig. 1.**
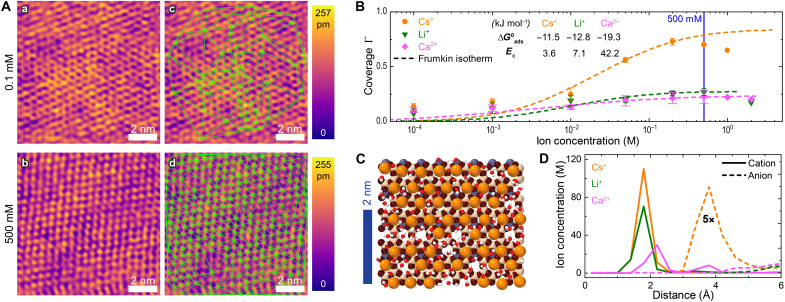
Quantification of interfacial thermodynamics from AFM micrographs and MD simulations. (**A**) AFM images of mica exposed to (a) 0.1 mM and (b) 500 mM CsNO_3_. Images in (c) and (d) refer to the same images in (a) and (b), respectively, with Delaunay triangulation patterns (green lines) overlaid. (**B**) Surface coverages Γ estimated from AFM images versus ion concentration (symbols) and their fitted Frumkin adsorption isotherms (dashed lines). $\Delta G_{\text{ads}}^{{}^{\circ}}$ and *E*_c_ are the fitting parameters (details in text S1). (**C**) MD snapshot of mica exposed to a Cs^+^ solution at 500 mM bulk concentration. (**D**) Interfacial ion concentration profiles versus distance from the surface from MD simulations. Anion profiles (Cl^−^) were scaled 5× for clarity.

The populations of ions adsorbing on charged surfaces can be visualized in situ with remarkable molecular-level resolution using AFM (*26*, *27*). In Fig. 1A, we quantitatively imaged ion-resolved and concentration-dependent adsorption of Cs^+^ ions on the highly negatively charged (001) plane of muscovite mica (Li^+^ and Ca^2+^ shown in fig. S1). The hexagonal structure of (001) mica is well visible at low ion concentrations, while the number of adsorbed ions increases with increasing ionic strength. In Fig. 1B, we further derived surface coverages (Γ), using an analysis protocol (reported in text S1 and fig. S2), which enables visualization of adsorbed ions as Delaunay network vertices [green lines, Fig. 1A (c and d) and further in fig. S3]. Since surface charge compensation is expected at a coverage of Γ = 0.5 (*28*), an ion-specific overadsorption regime (Γ > 0.5) was observed for Cs^+^, whereas Li^+^ adsorbs at Γ < 0.5. Since Ca^2+^ carries a twofold charge (per ion) compared to Cs^+^ and Li^+^, coverage at Γ ≈ 0.25 indicates surface charge compensation, with the experimental error bar denoting possible (slight) overadsoprtion.

This directly visualized coverage can be fitted well to a Frumkin isotherm for all ions (*19*, *29*, *30*). The fitting parameters are adsorption Gibbs free energy $\Delta G_{\text{ads}}^{{}^{\circ}}$ and lateral correlation energy *E*_c_, quoted in Fig. 1B.

As expected, adsorption Gibbs free energies are negative, owing to the negatively charged surface attracting the positively charged ions. In detail, the two single charged ions exhibit approximately half the $\Delta G_{\text{ads}}^{{}^{\circ}}$ observed for Ca^2+^, as expected for Coulomb interactions. *E*_c_ is lowest for Cs^+^, indicative of a low in-plane penalty for interfacial Cs^+^ adsorption. This is consistent with the surface overadsorption of Cs^+^ ions, although the adsorbate-adsorbate in-plane interaction is repulsive for all ions.

The ion-specific surface coverage (Fig. 1B) agrees well with the results from all atom classical MD simulations at 500 mM for all ions. In particular, simulated exposure of mica to Cs^+^ predicts overadsorption (see Fig. 1C), while exposure to Li^+^ and Ca^2+^ shows considerably weaker ion adsorption (see also fig. S1). Distance-dependent ion concentrations (Fig. 1D) indicate an enhanced Cs^+^ concentration close to the surface, and a slight consequent anion level rise (five times for better visibility). This anion distribution derives from Cs^+^ ions overscreening the mica surface charge, and consequent charge reversal in the first ion layer. Contrarily, Li^+^ shows close to neutralized screening. Ca^2+^, on the other hand, has a more complex adsorption structure with one ion layer at 2.2 Å, and the other at 3.9 Å, consistent with the presence of inner- and outer-sphere complexes observed elsewhere for other divalent ions (*31*). Coherent with AFM-derived isotherms, analysis of the net charge variations versus distance (see fig. S4A) further confirms slight overscreening for Ca^2+^ when considering both inner- and outer-sphere species.

### Ion-specific water structure at the interface

As demonstrated above, MD simulations reproduce the experimentally observed adsorption patterns. Additional Bader charge analysis, based on density functional theory (DFT) calculations of the solid-liquid interface, shows that the ionic charges of adsorbed Cs^+^, Li^+^, and Ca^2+^ are close to their expected formal valencies (see fig. S5). Hence, chemical bonding and charge transfer do not play a decisive role at the interface. Consequently, ion-water and water-water pair correlation functions (PCFs) were sampled from extensive MD simulations, considering both radial (RDFs) and orientational distribution functions (ODFs; see text S2 for details and fig. S6 for the PCF definition) (*25*, *32*, *33*).

**Fig. 2.**
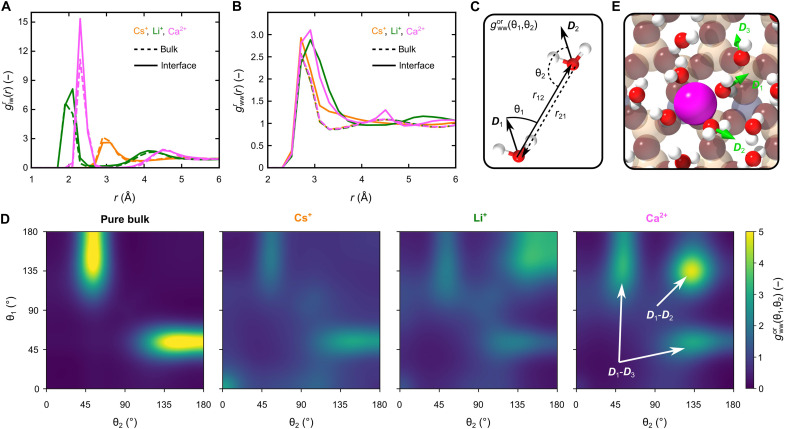
Adsorbed Cs^+^ maintains bulk water structure at the interface. Comparison of (**A**) ion-water $g_{\text{iw}}^{r}\left(r\right)$ and (**B**) water-water $g_{\text{ww}}^{r}\left(r\right)$ RDFs in the bulk and at the interface for Cs^+^, Li^+^, and Ca^2+^ 500 mM bulk solutions. We consider that the interface is up to 4 Å away from the surface. (**C**) Angles (θ_1_, θ_2_) for the sampled ODFs shown in (**D**) of pure bulk water and the electrolyte solutions at the interface. The intensity depends on the number of formed water pairs. Therefore, an overall intensity loss is observed for all interfacial ions. (**E**) MD snapshot of the emerging water structure for adsorbed Ca^2+^. The characteristic dipole configurations are indicated as green arrows for ion-bonded (*D*_1_-*D*_2_) and hydrogen-bonded (*D*_1_-*D*_3_) water, as observed in the Ca^2+^ ODF.

Figure 2A shows both bulk and interfacial ion-water RDFs $g_{\text{iw}}^{r}\left(r\right)$ for Cs^+^, Li^+^, and Ca^2+^. Upon adsorption, Li^+^ and Ca^2+^ ions increase their coordination with water compared to the bulk, while the relatively large Cs^+^ ion effectively repels water molecules from its shell. As the hydration shell radius remains constant for each ion, an increase in the hydration density is observed for Ca^2+^ and Li^+^ at the interface, and a decrease for Cs^+^ (see table S1). This dehydration behavior at the interface is a direct consequence of the relatively weak binding of hydration-shell water molecules of Cs^+^, evident from the comparable maxima of the respective $g_{\text{iw}}^{r}\left(r\right)$ (Fig. 2A) and the water-water RDF $g_{\text{ww}}^{r}\left(r\right)$ in Fig. 2B. We therefore classify Cs^+^ as a weakly hydrated ion, in contrast to Li^+^ and Ca^2+^, for which the first maxima of $g_{\text{iw}}^{r}\left(r\right)$ remarkably exceed those of $g_{\text{ww}}^{r}\left(r\right)$, indicating a strongly bound hydration shell. In addition to changes in the ion-water interaction, a considerable restructuring of water-water pairs is observed upon adsorption. As shown in Fig. 2B, the broadening of the first peak in $g_{\text{ww}}^{r}\left(r\right)$ at around 2.9 Å relates to a dense and increasingly structured water network being established for Li^+^ and Ca^2+^.

Figure 2D shows calculated dipole-dipole ODFs $g_{\text{ww}}^{\text{or}}\left(\theta_{1},\theta_{2}\right)$ for pure bulk water and interfacial water with solvated Cs^+^, Li^+^, and Ca^2+^ ions as defined in Fig. 2C. In bulk, the distribution maximum is approximately at (50°, 150°), corresponding to molecules acting as hydrogen-bond donors and acceptors. Qualitatively, the orientational configurations of adsorbed Cs^+^ resemble those of pure bulk water, indicating that hydrogen bonding between water molecules prevails. In contrast, driven by strong ion-dipole interactions, Li^+^ and Ca^2+^ orient water molecules into dipole-dipole configurations that disrupt the inherent tetrahedral structure, promoting dense interfacial water packing. The effect is associated with the emergence of new configurations for large (θ_1_, θ_2_), where water molecules are forced to align their dipoles opposite to typical hydrogen bonding. Further, the more pronounced peak for Ca^2+^ around (135°, 135°) can be attributed to stronger ion-dipole interactions for Ca^2+^ compared to Li^+^. This highly structured hydration shell of an adsorbed Ca^2+^ is evident from Fig. 2E, a characteristic MD snapshot. Therein, the green arrows indicate the dipole configurations for ion-bonded (*D*_1_-*D*_2_) and hydrogen-bonded (*D*_1_-*D*_3_) water in the Ca^2+^ ODF. Comparison of the remaining ODFs contained in the water-water PCF in fig. S7 further confirms that the enhanced orientational water structure at the interface has the trend Cs^+^ < Li^+^ < Ca^2+^. For detailed interpretation of the ion-water and water-water PCFs, see also text S7.

This remarkably altered interfacial water structure for Li^+^ and Ca^2+^ is also reflected in the three-body-angle distributions, shown in fig. S8 and the text S3. For these ions, in contrast to Cs^+^, interfacial water shows considerably reduced tetrahedral alignment (*34*), indicating that dipole-dipole interactions of water are effectively overcome by the ions’ strong fields.

### Water layering and polarization at the interface

**Fig. 3.**
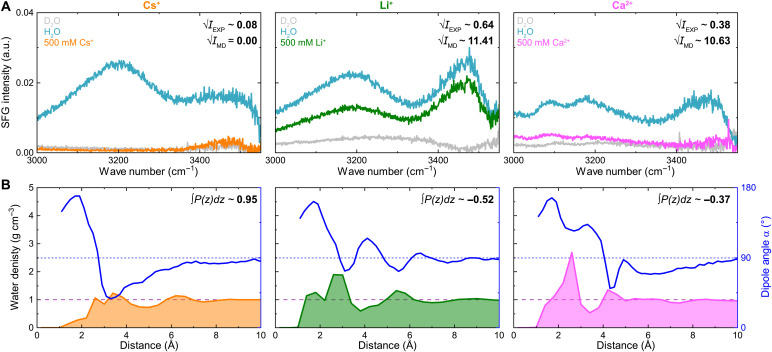
Ion-specific modulation of interfacial water structure and polarization revealed by SFG and MD. (**A**) SFG spectra of the OH stretching vibrations (~3000 to 3500 cm^−1^) on mica sequentially exposed to D_2_O (gray line), pure H_2_O (blue line), and a solution containing 500 mM of Cs^+^ (orange), Li^+^ (green), and Ca^2+^ (magenta). $\surd I_{\text{EXP}}$ corresponds to the square root of the normalized total measured signal intensity, whereas $\surd I_{\text{MD}}$ indicates the square root of the signal intensity estimated from MD results (details in text S6). (**B**) Water density profiles (shaded areas) and water dipole angle α (blue lines) calculated from MD simulations at 500 mM ion concentration as a function of the distance from the mica surface. The horizontal lines indicate the water bulk density (1 g cm^−3^, dashed) and the bulk dipole orientation of 90° (i.e., random orientation, dotted). The insets indicate the integral of the total polarization density *P*(*z*) from the mica surface to the bulk (details in text S6).

To further confirm the effect of Cs^+^, Li^+^, and Ca^2+^ on the interfacial water structure, we performed SFG spectroscopy in the frequency region of the OH-stretch vibrations (3000 to 3550 cm^−1^). SFG spectroscopy probes exclusively interfacial water, for which the inversion symmetry is broken. The corresponding SFG spectra are shown in Fig. 3A, with phase-resolved data in fig. S9 (see texts S4 and S5 and figs. S10 and S11 for experimental details and analysis). The different magnitudes of the mica-H_2_O and mica-D_2_O spectra arise mainly from different mica substrate thicknesses (see text S4). The mica-H_2_O interface exhibited much higher intensity than mica-D_2_O, which has no vibrations in this region (*35*), reflecting net polarization of interfacial H_2_O due to the negatively charged mica (*36*). The experimentally observed integrated SFG intensities $\surd I_{\text{EXP}}$ for mica against 500 mM salt solutions are quoted in Fig. 3A (top right). The intensity decrease relative to pure water follows Li^+^ < Ca^2+^ < Cs^+^, attributed to ion-specific adsorption (*37*–*39*). A smaller signal for Cs^+^ compared to Li^+^ was also seen for negatively charged silica interfaces (*40*).

The SFG intensity is typically interpreted to be proportional to the net orientation and density (i.e., polarization density *P* shown in fig. S4B) of interfacial water molecules. Therefore, we further compare the experimental results to the water density and average water orientation profiles obtained from MD simulations shown in Fig. 3B. First, the density data illustrate that, for systems with adsorbed Cs^+^, there is a notable depletion of water molecules within the first 2 to 3 Å from the surface, while Li^+^ and Ca^2+^ ions carry notable amounts of water to the interface, in line with observed SFG intensity tendencies. Water depletion is also consistent with observed attractive force profiles recorded for Cs^+^ (*41*–*43*), where a complete absence of hydration structuring was suggested (*44*). Second, in bulk water, random orientation gives an average dipole angle of 90°. At the negatively charged mica surface, water H-atoms face the surface (α > 90°) within 3 Å for all ions, but orientation profiles diverge beyond this region. Overscreening by Cs^+^ flips water molecules (α ≈ 45°), whereas Li^+^ nearly neutralizes the surface, yielding orientations fluctuating around 90°. For Ca^2+^, incomplete screening maintains a H-down orientation until the second ion layer introduces a net positive charge, after which the profile relaxes to bulk-like 90°.

The evaluated ∫P(z)dz, attributed to the SFG intensity, is quoted in Fig. 3B (top corners). The larger absolute value for Cs^+^ compared to Li^+^ and Ca^2+^ is not directly in line with the experimental SFG observations. Only by considering the electronic polarization effects of water molecules by the interfacial electric field (*38*, *45*), which does not result in a change of orientation, experimental SFG intensity trends can be reproduced (details in text S6). The corresponding estimated SFG intensity values from the MD data $\surd I_{\text{MD}}$ are further quoted in Fig. 3A, with the MD-based intensity ratio Li^+^/Ca^+^ ≈ 1.1 being only slightly smaller than the experimental ratio of 1.7. We can hence conclude that the weak signal in Cs^+^ spectra is not simply linked to the low water density (i.e., depletion), as one may expect. The spectral response is moderated by third-order effects, with local electric fields electronically polarizing the water molecules at the interface.

### Interplay between enthalpic and entropic contributions

In the present work, PCFs are used as quantitative descriptors for ion hydration, and, as such, they provide a basis for a quantitative evaluation of interfacial thermodynamics via their correlation with entropic contributions. Therefore, we further calculate ion-water and water-water contributions to the standard entropy of ion solvation Δ*S*_tot_ (*46*) in the bulk and at the interface to estimate entropic penalties of adsorption directly from PCFs (details in texts S2 and S7 and figs. S12 and S13).

Table S2 summarizes the calculated translational and orientational entropic contributions of ion-water and water-water interactions. The calculated bulk entropy values quantitatively reveal a clear separation between weakly and strongly hydrated ions, with Cs^+^ exhibiting a reduced entropic penalty compared to Li^+^ and Ca^2+^. Our results further show that orientational entropy is the major contributor to the thermodynamics of both interaction types at the interface. This extends the well-established importance of orientational correlations in bulk water (*47*, *48*), for water contact angles (*49*), and in bulk electrolytes (*25*), to electrolyte systems at interfaces, indicating that the inner double layer cannot be accurately described without accounting for orientational entropic contributions. Although both water-water and ion-water correlations add to the overall entropic penalty of ion adsorption, the latter are significantly smaller, highlighting that perturbations in the water-water network dominate the interfacial entropy change. Quantitatively, Cs^+^ experiences the smallest total entropic penalty among all ions—the weak ion-dipole interaction renders the water structure unaltered (i.e., bulk-like). In contrast, Li^+^ and Ca^2+^ induce substantial ordering to interfacial water molecules and, consequently, larger changes in entropy.

Ion adsorption is further controlled by the counterbalance of the entropic penalties and ion-surface (*W*_ion-surf_), ion-dipole (*W*_ion-dipole_), and ion-ion (*W*_ion-ion_) interactions. We approximate these interactions for a given surface concentration using a classical model of pairwise electrostatic interactions at the interface (details in text S8). In this model, the effective interfacial dielectric constant ε is the only unknown variable. All other parameters (e.g., pairwise distances, dipole orientations, and interfacial water density) are fixed by atomistic simulations or experimental observations from subnanometer AFM imaging (details in text S8). Thereby, we bring molecular resolution to this thermodynamic modeling. Further, using the equilibrium condition

$$\Delta G=0=\sum_{i}W_{i}\left(\varepsilon \right)-T\;\Delta S$$ (1)

We can solve for the energetic contributions by extracting ε as a fitting parameter, implicitly accounting for structural changes to the interfacial water environment by ion-specific adsorption.

**Fig. 4.**
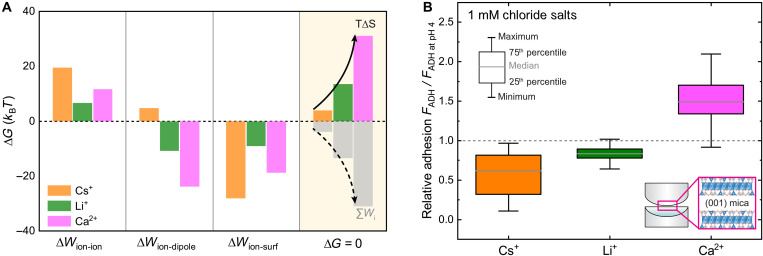
Ion-specific entropic penalties determine variations of interfacial dielectric screening and control adhesion properties between two surfaces. (**A**) Energetic contributions to interfacial structure formation at equilibrium calculated from a summation of interfacial pair interactions (compare text S8). (**B**) SFA measurements of cation-dependent adhesion between two (001) muscovite mica surfaces. Box and whiskers plot shows pull-off adhesion force for each 1 mM chloride salt with combined measurements from five independent experiments, normalized by the average adhesion measured in pH 4 HCl solution as described in Materials and Methods.

Figure 4A shows the obtained contributions of pairwise interactions in 500 mM solutions. First, in-plane *W*_ion-ion_ interactions are generally repulsive and strongest for Cs^+^ with the highest coverage, and hence, shortest average in-plane distances. Second, due to the increased hydration of adsorbed Li^+^ and Ca^2+^, *W*_ion-dipole_ interactions are exothermic, indicating favorable surface interactions. Contrastingly, Cs^+^ ions pay an energetic penalty for their dehydration upon adsorption. Together, these two contributions would, in principle, not support high adsorption of Cs^+^.

However, the attractive ion-surface interaction *W*_ion-surf_ for Cs^+^ is considerably larger compared to those of Li^+^ and Ca^2+^ and can overcome the repulsive lateral interaction arising from the high Cs^+^ coverage. This is directly related to the considerable depletion of interfacial water and the consequent decrease in dielectric screening, i.e., by a lower ε. The derived values of ε are 88, 85, and 50 for Li^+^, Ca^2+^, and Cs^+^, respectively. For Cs^+^, ion-surface attractions are hence considerably increased, while those of Li^+^ and Ca^2+^ are decreased owing to the higher interfacial water density.

Thus, our data quantitatively show that the interplay of low entropic penalty and high interfacial adsorption energies due to the low dielectric screening favors inner-sphere overadsorption of Cs^+^. This is due to the weak Cs^+^-water interaction, driving interfacial water depletion. Conversely, small and/or highly charged ions such as Li^+^ and Ca^2+^ strongly align water dipoles, causing large entropic penalties and consequently enhanced dielectric screening. This presents a far more complex picture than the established Hofmeister concept, but ultimately derives from the strength of ion-water interactions. As shown in Fig. 4B, this manifests in distinct adhesion trends measured using a surface force apparatus (SFA): Cs^+^ yields low adhesion due to overadsorption and consequent strong electrostatic repulsion at water-depleted interfaces. Li^+^ resembles baseline screening, and Ca^2+^ enhances adhesion via depletion of entropically unfavorably confined water, leading to bridging interactions (*50*–*52*). This is also visible in a rather small decrease of the adhesive interaction distance, which characterizes the thickness of the confined electrolyte (i.e., the hardwall distance in fig. S14B).

## DISCUSSION

Overall, we presented a combined experimental and theoretical model with atomic-level detail that provides a well-grounded framework to interpret ion-specific effects at solid-liquid interfaces, mostly determined by the fully quantifiable strength and orientational distribution of ion-water interactions. Based on our findings, we identify the key requirements leading to (over)adsorption in an energetic balance between the following ingredients: (i) the existence of a sufficiently strong surface-ion driving force (electrostatic and/or other interactions), balanced by (ii) ion-ion correlations in the adsorbed layer, and (iii) an interfacial solvent response that modifies the effective screening (including dielectric reduction and the associated entropic cost or gain from restructuring water).

As such, the model proposed does not impose inner- or outer-sphere adsorption (and thereby water depletion) as necessary conditions for overadsorption. Rather, it sheds light on the molecular-level driving forces leading to inner- or outer-sphere adsorption within the same unified view. Namely, we observe that (i) inner-sphere overadsorption is strongly coupled to interfacial water depletion and dielectric reduction. This behavior is typically linked to weakly hydrated ions [e.g., Cs^+^ and Rb^+^; (*30*, *53*)] that impose low entropic penalties on the water structure. (ii) On the other hand, overadsorption driven by strongly correlated outer-sphere species such as Ca^2+^ is driven by stronger ion-surface interactions (here, electrostatics), while substantial overadsorption is limited by the consequential highly ion-pinned water structure. This applies to strongly hydrated ions [e.g., Ca^2+^ and other multivalent ions in general (*54*, *55*)], whose strong electric fields induce considerable entropic costs for water restructuring, which is balanced by higher surface-ion interactions, while not depleting water from the interface region.

Ultimately, the thermodynamic framework proposed in this work is envisaged to find applicability for a variety of other surface chemistries and conditions, where understanding of interface thermodynamics is pivotal in demistifying complex interfacial mechanisms (*56*). For other surface types, additional contributions may need to be considered, e.g., image-charge effects on metallic surfaces (*57*), or variations of surface charges due to adsorption-dependent protonation-equilibria on pH-responsive surfaces (*58*, *59*), etc. The relative importance of each contribution may vary specifically for each studied system, although their physical nature remains unaltered and well resolvable.

In summary, molecular-resolution AFM imaging combined with concentration-dependent adsorption isotherms, MD simulations, and SFG spectroscopy provide a fully quantitative picture of interfacial ion adsorption. We show that disruption or depletion of interfacial water structure imposes distinct entropic penalties and dielectric responses that govern ion-surface interactions, as well as macroscopic behavior in applications. SFG spectroscopy further reveals that these structural changes modify the local electric polarization of water, directly linking interfacial structuring to dielectric modulation, along with density variations derived from MD simulations. Together, these results establish a thermodynamically grounded framework in which water controls ion-specific adsorption through the interplay of entropy, dielectric screening, and polarization. Our approach enables a consistent understanding of inner double-layer effects and structuring at solid-liquid interfaces, with broad applicability, by offering a clear, thermodynamically grounded first principles understanding.

## MATERIALS AND METHODS

### Materials

High-purity, optical-grade ruby muscovite mica was supplied by S&J Trading Inc., USA. All chemicals mentioned further in the next sections were purchased in high-purity (American Chemical Society grade) and used without further purification. All the solutions were prepared using ultrapure deionized MilliQ water (resistivity of 18.2 MΩ cm^−1^).

### Atomic force microscopy

Mica sheets (~2 cm by 2 cm) were glued using ultraviolet-curing glue (NOA 81, Norland) to magnetic disks as support and cleaved along the {001} planes before every experiment. The freshly cleaved mica was promptly exposed to the cation-rich solution at a certain concentration (~0.2 ml). Before imaging, the system was allowed to equilibrate for ~10 min (i.e., under immersion in the solution). The solutions were prepared by diluting CsNO_3_, LiNO_3_, and CaCl_2_ in water to obtain concentrations ranging from 0.1 to 2000 mM.

Highly resolved AFM was achieved using dynamic imaging (i.e., amplitude modulation). Gold-coated cantilevers (Arrow-UHFAuD, NanoWorld) were tuned and approached to the mica surface, while the latter was still immersed in the liquid solutions of different ionic strengths. This normally caused a shift of the nominal resonance frequency (2 MHz, measured in air) to lower resonance values (~600 to 1000 kHz). Quasi–molecular-level resolution was possible by photothermally exciting the cantilevers in amplitude modulation. Typical oscillation amplitudes far from the surface ranged between 0.3 and 1.2 nm. All the experiments were conducted within a CypherES AFM (Oxford Instruments). Topography maps of 10 nm by 10 nm were obtained with a scan frequency of 5 to 7 Hz. The best resolutions could be achieved by imaging in a slightly repulsive mode. Adsorption of imaged ions on mica was quantified with a dedicated protocol (*12*) and modeled with Frumkin isotherms, both described in the text S1.

### Phase-resolved and conventional SFG spectroscopy

The laser setup was constructed similar to a report in literature (*60*). The laser source for SFG spectroscopy was a Ti:sapphire amplifier (Astrella, Coherent) with a 1-kHz repetition rate, ~30-fs pulse duration, and an output pulse energy of 7 mJ. The 800-nm output pulse was split into several parts. Two millijoules was used to generate the infrared beam for the experiments using an optical parametric amplifier (TOPAS PRIME, Light Conversion) with a successive noncollinear difference frequency generation stage (Light Conversion). The resulting infrared beam had an energy of about 6 μJ and was centered at 3100 nm with a full width at half maximum (FWHM) of about 340 cm^−1^. The 800-nm beam was narrowed down by sending 1 mJ of the laser output through a homemade 4f pulse shaper in reflection configuration, yielding pulses with an FWHM of about 20 cm^−1^ and an energy of 20 μJ. The narrowed 800-nm and infrared pulses were focused onto the local oscillator generator with a fused silica lens (LA4716-B, Thorlabs) and a CaF_2_ lens (LA5042, Thorlabs), respectively. The local oscillator generator consisted of a 150-nm-thick sputtered ZnO layer on top of a CaF_2_ substrate (1-mm thickness, 25-mm diameter, Crystal GmbH). The 800-nm, infrared and local oscillator sum frequency pulses were collimated by a 60° off-axis parabolic mirror (MPD246-P01, Thorlabs). Afterward, the local oscillator sum frequency light was passed through a 2-mm thick CaF_2_ plate to induce a phase delay. All three beams were refocused onto the sample by a 60° off-axis parabolic mirror (MPD246-P01, Thorlabs). The incident angles for the 800 nm and infrared beam were ~60° and ~40° with respect to the sample surface normal, respectively. All measurements were done with s-polarization for the 800 nm and p-polarization for the infrared light. Both sum frequency pulses from the local oscillator generator and the sample were detected in s-polarization.

The mica substrates were mounted on a stainless-steel flow cell, which allowed exchange of the solution beneath the mica substrate while staying on the same spot of the substrate surface. A new sample was freshly cleaved before each measurement series, allowing measurements on pristine surfaces. The thickness of the cleaved samples was between 140 and 176 μm. In each measurement series, the freshly cleaved mica surface was first measured in contact with D_2_O (99.9%, Deutero GmbH), then ultrapure H_2_O, and, last, the respective 500 mM CsNO_3_, LiNO_3_, or CaCl_2_ salt solution. The reflected sample and local oscillator sum frequency light was collimated and refocused onto the slit of a spectrometer (Andor Shamrock 303i, Oxford Instruments) where the beams were dispersed by a 1200 grooves mm^−1^ grating and subsequently detected with a charge-coupled device image sensor (Andor Newton EMCCD, Oxford Instruments) at −75°C. The obtained interference signals were processed as described in literature (*61*). Briefly, the spectrum was inverse Fourier-transformed into the time domain, where one of the cross-terms was selected using a filter function. Next, the cross-term was Fourier-transformed back to the frequency domain. The same procedure was done for the reference, which was a z-cut optically polished quartz cube. Afterward, the processed sample spectrum was divided by the processed spectrum of the reference. Measurements of mica samples in contact with D_2_O were used to correct for the different phase shift of the local oscillator sum frequency signal with the sample and quartz reference sum frequency signals. After each phase-resolved measurement (data shown in fig. S9), a conventional/homodyne detected SFG spectrum (data shown in Fig. 3B) was also acquired by blocking the local-oscillator SFG light in between the parabolic mirrors. The homodyne spectrum was background-corrected and normalized with the homodyne SFG signal of the quartz reference. All experiments were carried out at ambient pressure in a temperature-controlled laboratory at 20°C.

### Surface force apparatus

Forces between two atomically smooth (001) mica surfaces were measured using the in-house modified SFA, equipped with a strain gauge-type force sensor as described by Wieser *et al.* (*62*). Mica was freshly cleaved along (001) crystallographic planes to a uniform thickness of several micrometers, cut with sharp surgical scissors, back-coated with 45 nm of Au for the SFA-coupled white light interferometry, and glued to cylindrical glass disks with a radius of curvature of 2 cm using EPON 1004 hot-melt epoxy resin (supplied by SurForceLLC, USA), following the standard mica preparation protocols (*63*–*65*). The two cylinders were then crossed at ~90° to yield a spherical contact area. In this work, the SFA experiments were designed to compare cation-dependent adhesion between mica surfaces. We used seven chloride salt solutions: LiCl, NaCl, KCl, CsCl, MgCl_2_, CaCl_2_, and SrCl_2_. Forces were measured as a function of surface separation and adhesion, and hardwall contact position parameters were extracted from each measured force curve. The forces were normalized with the radius of curvature of the SFA support disks (*R*_c_ = 0.02 m). We define adhesion as a pull-off force (maximum negative force) measured on separating the two surfaces, just before the adhesive jump-out from the contact. Hardwall position is defined as an average surface separation at a load range fixed for each of the six separate experiments. In each experiment, for all tested chloride salt solutions, we followed adhesion between the same pair of mica surfaces and in the same contact region to avoid any effects of a varying contact shape, contact area, and mica heterogeneity on the measured adhesion. At the beginning of each experiment and after each chloride salt solution, we measured forces in pH 4 HCl solution. The pull-off adhesion measured in chloride salt solutions was then normalized by dividing it by the average pull-off adhesion measured in pH 4 HCl directly after a given chloride salt. Similarly, the hardwall position in a given chloride salt was reported as a hardwall position shift with respect to the average hardwall position measured in pH 4 HCl (as a difference) directly after a given chloride salt. We only used the experiments, in which the reference pull-off force measured in pH 4 HCl did not change significantly throughout a given experiment. Force run data were analyzed in the SFA explorer software (*66*). Thicknesses of micas were calculated from images of two micas in contact in MilliQ water at the beginning of each experiment.

### MD simulations

MD simulations were performed on a three-dimensional model system consisting of aqueous electrolytes confined between two negatively charged muscovite mica surfaces. The mica models, based on crystallographic x-ray diffraction data (*67*), were constructed using the unit cell formula KAl_2_(Si_3_Al)O_10_(OH)_2_. Each mica surface comprised 32 unit cells (8 by 4) with lateral dimensions of 4.16 nm by 3.61 nm, and the separation distance between the two surfaces was chosen as 6 nm. This distance is sufficiently large to ensure a bulk liquid behavior at the center of the channel. Within each tetrahedral layer of mica, one silicon (Si) atom per unit cell was substituted by an aluminum (Al) atom via isomorphic substitution, resulting in a net negative surface charge. The substitution was implemented in an orderly manner over the whole mica surface, corresponding to a surface charge density of −0.33 C m^−2^. As the electrolyte, we used chloride (Cl^−^) solutions of cesium (Cs^+^), lithium (Li^+^), and calcium (Ca^2+^) ions. For each ion type, the number of salt ions and water molecules in the simulation box was always adjusted to maintain a bulk ionic concentration of 0.5 M and a bulk water density of 1000 kg m^−3^. This ensures a consistent reference state that allows direct comparison of the different ion types under the same thermodynamic state and ionic conditions. Note that the high ionic concentration is often necessary in MD simulations to achieve an enhanced statistical sampling of atomic structures and dynamics. To maintain overall electroneutrality in the simulation box, an excess of cations was added to balance the negative surface charge of the mica surfaces. The simulations were performed using the open-source Large-Scale Atomic/Molecular Massively Parallel Simulator (LAMMPS) software package (*68*). Interatomic interactions were described based on the CLAYFF model for mica (*69*) and SPC/E model for water (*70*). Ion interactions were modeled using the parametrization studies by Joung and Cheatham (*71*) and by Mamatkulov *et al.* (*72*). These models can accurately reproduce physical properties including hydration Gibbs energy, solubility of salts, and pair correlations (*71*–*73*). Interactions between dissimilar atoms were calculated using the Lorentz-Berthelot mixing rule. To enforce the rigidity of the water molecules, the SHAKE algorithm was applied to constrain bond lengths and angles (*74*). The atomic coordinates of the mica surfaces remained fixed during the simulations. Periodic boundary conditions were implemented along the *x* and *y* axes, while the *z* axis was confined by channel walls. To handle electrostatic interactions in the surface normal direction with a reduced periodicity, a slab correction was incorporated into the Ewald summation technique (*75*). Long-range electrostatic forces among charged species were evaluated using the particle-particle-particle-mesh (pppm) method, maintaining a root mean square accuracy of 10^−5^. A cutoff distance of 1.1 nm was applied uniformly to both Lennard-Jones and Coulomb potentials. Newton’s equations of motion were integrated using the Verlet algorithm using 1-fs time step. Temperature and pressure were kept constant at *T* = 300 K and *p* = 1 atm, respectively, using the Nose-Hoover thermostat in an NVT ensemble. Initially, equilibrium simulations were performed for durations ranging from 20 to 35 ns, depending on the ion type. This variation in equilibration time arose from differences in the ion dynamics associated with ion size and charge density, as bulky but low charged density Cs^+^ requires longer time to reach an adsorption saturation at the surface. Subsequently, each simulation ran for an additional 10 ns for data collection. High statistical averaging was achieved using 15 independent simulations, each started from different initial configurations. MD simulations are used to quantify ion concentrations, densities, RDFs, PCFs, and entropic penalties.

### DFT-Bader charge analysis

Ab initio simulations of ion adsorption on muscovite mica were carried out with the Vienna Ab Initio Simulation Package (*76*, *77*) using the projector-augmented waves (*78*, *79*). Exchange and correlation were treated within the generalized gradient approximation with the Perdew-Burke-Ernzerhof (PBE) formalism (*80*) and D3 dispersion correction with zero damping (*81*). We used the recommended pseudopotentials for all elements, the cutoff energy was set to 500 eV, and the stopping criterion for electronic minimization was set to <10^−5^ eV. We used Γ-centered *k* meshes (*82*) with a 10 by 6 by 2 grid for the bulk structure with 84 atoms, and a 4 by 2 by 1 grid for the 2 by 2 by 1 supercell containing 336 atoms with a 20-Å vacuum added on top. We first relaxed the bulk structure with all degrees of freedom using the conjugate gradient algorithm, then built the 2 by 2 by 1 supercell with the negatively polarized aluminosilicate tetrahedrons facing up freely exposed, followed by 20 Å of vacuum, and relaxed the top three atomic layers (the top tetrahedrons), while keeping the rest of the positions as well as the simulation box fixed. After this step, we added water molecules in a 5-Å-thick layer above the mica slab for the initial positions, and two Ca or four Li or Cs atoms at the upper end of the water-occupied region. The number of water molecules was 24 for Ca and 36 for Li and Cs. We then relaxed the atomic positions again with the mica atomic positions frozen like before, observing migration of the ions toward the mica surface, while interacting with the water molecules. The simulations reached the stopping criteria when some of the ions came into direct contact with the mica slab, while other ions were fully hydrated. We then performed a Bader charge analysis (*83*, *84*) to study the charge distribution in the relaxed systems.

### Electrolyte structure and entropy analysis

To characterize the electrolyte structure, ion-water and water-water PCFs were sampled from MD trajectories. For the analysis of the orientational water structure in Fig. 2, the Kirkwood superposition approximation (*32*, *33*) was applied. Quantitative analysis of the ion-water and water-water structure is based on the homogeneous triplet ion-water-water correlation function (*33*) from which we further calculated the standard entropy of ion solvation (*46*) Δ*S*_tot_ in the bulk and at the interface. For detailed demonstrations, see text S2.

## Supplementary Materials

### This PDF file includes:

Supplementary Texts 1 to 8 Figs. S1 to S14 Tables S1 and S2 References
