# Supplementary material for: Entropic-dielectric interplay governs ion adsorption in inner electric double layers
Source: Sci Adv. 2026 May 15;12(20):eaee9469. doi: 10.1126/sciadv.aee9469 (PMC13178531; doi:10.1126/sciadv.aee9469)
Supplement: Supplementary file 1 — Supplementary Texts 1 to 8 Figs. S1 to S14 Tables S1 and S2 References [file sciadv.aee9469_sm.pdf]

Science Advances
AAAS

# Supplementary Materials for

## Entropic-dielectric interplay governs ion adsorption in inner electric double layers

Matteo Olgiati *et al.*

Corresponding author: Markus Valtiner, markus.valtiner@tuwien.ac.at

*Sci. Adv.* **12**, eaee9469 (2026)
DOI: 10.1126/sciadv.aee9469

**This PDF file includes:**

Supplementary Texts 1 to 8
Figs. S1 to S14
Tables S1 and S2
References

## Supplementray Text 1. Protocol of analysis for high resolution AFM

In this work, we used a semi-automated image analysis algorithm to quantify adsorption phenomena observed with AFM. Such protocol was already described in detail in previous publications (*12, 85*) and relies on Delaunay triangulations to visualize the ion positions and average nearest neighbor distances. Here, we further improved the method to describe the surface coverage ($\Gamma$).

In short, we define a plane (*AL*) in the z-direction (*i.e.*, perpendicular to the (001) surface) that describes the adsorption state on mica. Within this plane, we can count the individual ions ($N_{ion}$) and relate this number to the calculated total number of adsorption sites ($N_{sites}$) to express the surface coverage as follows:

$$\Gamma = \frac{N_{ion}}{N_{sites}}. \tag{S1}$$

To define the adsorption plane *AL*, we analysed the histogram's distribution. We assumed that the mode of the distribution ($\mu^*$) represents an average reference plane value for the mica substrate. Therefore, adsorbates are expected to sit on the lattice sites above such modal plane. We then defined a threshold that describes the adsorption layer (*AL*) through a certain displacement $\varepsilon$ from the modal plane (see also Fig. S2a):

$$AL = \mu^* + \varepsilon \tag{S2}$$

Further, a statistical description of $\varepsilon$ was achieved by relating it to the standard deviation ($\sigma$) of the distribution. For this purpose, we fitted each topography distribution with a skewed normal distribution function described by:

$$F(x) = 2\frac{A}{\sigma\sqrt{2\pi}}e^{-\frac{(x-\mu)^2}{2\sigma^2}}\left[1 + erf\left(\frac{\zeta(\frac{x-\mu}{\sigma})}{\sqrt{2}}\right)\right], \tag{S3}$$

where $A$ is the amplitude of the distribution, $\mu$ is the mean of the distribution, $\sigma$ is the standard deviation and $\zeta$ is a dimensionless parameter defining the skewness. If $\zeta < 0$, the distribution is said to be positively skewed, whereas if $\zeta > 0$, the distribution is said to be negatively skewed. When $\zeta = 0$, the distribution is normal.

We observed that $\zeta$ (*i.e.* the skewness) showed a certain trend as a function of the solution concentration (see Fig. S2b): $\zeta$ has (on average) values between -1 and 1, except for i) very diluted solutions of $Li^+$, where the distribution becomes positively skewed (*i.e.*, $\zeta$ < -1), and ii) for exposure to $Cs^+$-rich solutions, where the distribution becomes more significantly negatively skewed (*i.e.*, $\zeta$ > 1). For -1≤ $\zeta$ < 1 (blue shade in Fig. S2b), the distribution can be well approximated with a normal distribution function, as the mean and the mode almost coincide. On the other hand, for higher skewness ($\zeta$ < -1 and/or $\zeta \geq 1$, lilac area in Fig. S2b), the distribution cannot be equally well described by a normal distribution function, as the separation between mode and mean becomes larger. For this reason, we further inferred that the skewness of the distribution could be a descriptive parameter of the adsorption state, as the presence of a lower/higher population of adsorbate species could have a direct influence on how the distribution gets skewed (*i.e.*,

positively or negatively). A more pronounced negative skew ($\zeta > 1$) would be expected for highly covered surfaces, as more pixels are expected to populate the high topography region of the histogram. Conversely, less covered surfaces should show a lower population of pixels in the high topography region and thereby a more positively skewed distribution ($\zeta <$ -1).

Empirically, we found that $\varepsilon$ can be defined as:

- $2\sigma$ , when -1 $\leq \zeta <$ 1
- $\sigma$, when $\zeta <$ -1 or $\zeta \geq$ 1

This is a consequence of the fact that a highly skewed distribution systematically showed a larger $\sigma$.

Once *AL* is defined as per equation S2, the images are further analyzed as previously reported (*12*). In short:

- ROI maps containing the spatial coordinates of individual ions are generated in ImageJ through an automated-counter algorithm. In the present research, we further added size and circularity constraints (size > 4 pixels$^2$ and circularity > 0.8) in order to systematically eliminate outliers;
- We applied Delaunay triangulations in ImageJ in order to count the number of detected ions $N_{ion}$ and evaluate the average distance between them.

Quantification of $N_{ion}$ allowed us to express the coverage $\Gamma$ as per equation S1.

Cation adsorption was further modelled as a function of the ion concentration using a Frumkin-modified competitive adsorption isotherm described elsewhere (*29*) and by the following equation:

$$\Gamma_{\mathrm{Me^{n+}}} = \frac{\gamma K_{\mathrm{Me^{n+}}} C_{\mathrm{Me^{n+}}} exp(-\frac{E_c}{RT}\Gamma_{\mathrm{Me^{n+}}})}{1 + K_{\mathrm{Me^{n+}}} C_{\mathrm{Me^{n+}}} exp(-\frac{E_c}{RT}\Gamma_{\mathrm{Me^{n+}}}) + K_{\mathrm{H_3O^+}} C_{\mathrm{H_3O^+}}}, \tag{S4}$$

where $\mathrm{Me^{n+}}$ represents the cation species adsorbed on mica, $\gamma$ the maximum coverage, $K$ the Langmuir equilibrium constants, $C$ the molar concentration, $E_c$ the adsorbate-adsorbate correlation energy term, $R$ and $T$ the molar gas constant and temperature, respectively. The value of $K_{\mathrm{H_3O^+}}$ was taken from (*29*) and $C_{\mathrm{H_3O^+}}$ was obtained by pH determination of each diluted electrolyte (pH 5-6). The standard Gibbs free energy of adsorption was further estimated as:

$$\Delta G^0_{ads} = -\mathrm{R}T \ln K_{\mathrm{Me^{n+}}}. \tag{S5}$$

## Supplementray Text 2. Electrolyte structure and entropy from pair correlation functions

According to Lazaridis (*32*), the standard entropy of ion solvation(*46*) $\Delta S_{\mathrm{tot}}$ can be partitioned into an ion-water $S_{\mathrm{iw}}$ and water-water $\Delta S_{\mathrm{ww}}$ interaction term,

$$\Delta S_{\mathrm{tot}} = S_{\mathrm{iw}} + \Delta S_{\mathrm{ww}} . \tag{S6}$$

For a given system of an infinitely dilute electrolyte, the terms in Eq. (S6) have the form

$$S_{\mathrm{iw}} = -\frac{\mathrm{R}\, n_0}{\Omega} \int g_{\mathrm{iw}}(\mathbf{r}, \omega) \ln g_{\mathrm{iw}}(\mathbf{r}, \omega)\, \mathrm{d}\mathbf{r}\mathrm{d}\omega \,, \tag{S7}$$

$$\begin{aligned} S_{\mathrm{ww}} = \Delta S_{\mathrm{ww}} + S^{0}_{\mathrm{ww}} = -\frac{\mathrm{R}\, n_0^2}{2\,\Omega^2} \int g_{\mathrm{iw}}(\mathbf{r}, \omega) g_{\mathrm{iw}}(\mathbf{r}', \omega') \times \\ \{g^{\mathrm{inh}}_{\mathrm{ww}}(\mathbf{r}, \mathbf{r}', \omega, \omega') \ln g^{\mathrm{inh}}_{\mathrm{ww}}(\mathbf{r}, \mathbf{r}', \omega, \omega') - g^{\mathrm{inh}}_{\mathrm{ww}}(\mathbf{r}, \mathbf{r}', \omega, \omega') + 1\}\, \mathrm{d}\mathbf{r}\mathrm{d}\mathbf{r}'\mathrm{d}\omega\mathrm{d}\omega' \,. \end{aligned} \tag{S8}$$

Here, R is the gas constant, $\mathbf{r}$ and $\mathbf{r}'$ denote the position of two water molecules with respect to the ion, and $\omega$ and $\omega'$ denote the orientation of these water molecules with respect to the ion in terms of the angles defined in Fig. S6a. $\Omega$ is the integral over $\omega$ and $n_0$ is the bulk water number density. $g_{\mathrm{iw}}$ denotes the ion-water correlation function, $g^{\mathrm{inh}}_{\mathrm{ww}}$ is the water-water pair correlation function (PCF) for the inhomogeneous system, *i.e.* for water containing dissolved ions, and $S^{0}_{\mathrm{ww}}$ denotes the entropy of pure bulk water. The ion-water and water-water correlation functions are related to the homogeneous triplet correlation function $g_{\mathrm{iww}}$, given that the ion sits at the origin $\mathbf{r}_{\mathrm{i}} = \mathbf{0}$,

$$g_{\mathrm{iww}}(\mathbf{0}, \mathbf{r}, \mathbf{r}', \omega, \omega') = g_{\mathrm{iw}}(\mathbf{r}, \omega) g_{\mathrm{iw}}(\mathbf{r}', \omega') g^{\mathrm{inh}}_{\mathrm{ww}}(\mathbf{r}, \mathbf{r}', \omega, \omega') \,. \tag{S9}$$

The calculation of $g_{\mathrm{iww}}$ from molecular simulations requires sampling an extremely large number of configurations, which leads to slow convergence of the results. To make the calculations feasible, we therefore assume spherical symmetry of water molecules around the ion, such that the correlation functions depend on the distance between the water molecule(s) and the ion only, *i.e.* $g_{\mathrm{iw}}(\mathbf{r}, \omega) = g_{\mathrm{iw}}(r, \omega)$ and $g^{\mathrm{inh}}_{\mathrm{ww}}(\mathbf{r}, \mathbf{r}', \omega, \omega') = g^{\mathrm{inh}}_{\mathrm{ww}}(r, r', \omega, \omega')$.

The ion-water and water-water entropy can further be partitioned into translational and orientational contributions by factoring the corresponding correlation functions into a radial and orientational part,

$$g_{\mathrm{iw}}(r, \omega) = g^{\mathrm{r}}_{\mathrm{iw}}(r)\, g^{\mathrm{or}}_{\mathrm{iw}}(\omega|r) \tag{S10}$$

$$g^{\mathrm{inh}}_{\mathrm{ww}}(r, r', \omega, \omega') = g^{\mathrm{r}}_{\mathrm{ww}}(r, r')\, g^{\mathrm{or}}_{\mathrm{ww}}(\omega, \omega'|r, r') \tag{S11}$$

where $g(\omega|r)$ denotes the conditional distribution function of finding a water molecule with orientation $\omega$ located at $r$, and $g^{\mathrm{r}}_{\mathrm{iw}}(r)$ and $g^{\mathrm{r}}_{\mathrm{ww}}(r)$ are the radial distribution functions given by

$$g^{\mathrm{r}}_{\mathrm{iw}}(r) = \frac{1}{\Omega} \int g_{\mathrm{iw}}(r, \omega)\, \mathrm{d}\omega \,, \tag{S12}$$

$$g^{\mathrm{r}}_{\mathrm{ww}}(r, r') = \frac{1}{\Omega^2} \int g_{\mathrm{ww}}(r, r', \omega, \omega')\, \mathrm{d}\omega\mathrm{d}\omega' \,. \tag{S13}$$

For the translational entropy due to ion-water correlations, $S^{\mathrm{tr}}_{\mathrm{iw}}$, and water-water correlations, $S^{\mathrm{tr}}_{\mathrm{ww}}$, it follows that

$$S^{\mathrm{tr}}_{\mathrm{iw}} = -2\pi \mathrm{R} n_0 \int \{g^{\mathrm{r}}_{\mathrm{iw}}(r) \ln g^{\mathrm{r}}_{\mathrm{iw}}(r) - g^{\mathrm{r}}_{\mathrm{iw}}(r) + 1\} r^2\, \mathrm{d}r \,, \tag{S14}$$

$$S^{\mathrm{tr}}_{\mathrm{ww}} = -8\pi^2 \mathrm{R} n_0^2 \int g^{\mathrm{r}}_{\mathrm{iw}}(r) g^{\mathrm{r}}_{\mathrm{iw}}(r') \{ g^{\mathrm{r}}_{\mathrm{ww}}(r,r') \ln g^{\mathrm{r}}_{\mathrm{ww}}(r,r') - g^{\mathrm{r}}_{\mathrm{ww}}(r,r') + 1\} r^2 r'^2 \,\mathrm{d}r\mathrm{d}r' \,. \tag{S15}$$

Based on the conditional distribution functions given in Eqs. (S10) and (S11), the orientational entropy of ion-water and water-water configurations can be evaluated as a kernel function $\mathcal{S}^{\mathrm{or}}$ that only depends on the ion-water distance,

$$\mathcal{S}^{\mathrm{or}}_{\mathrm{iw}}(r) = \frac{\mathrm{R}}{\Omega} \int g^{\mathrm{or}}_{\mathrm{iw}}(\omega|r) \ln g^{\mathrm{or}}_{\mathrm{iw}}(\omega|r) \,\mathrm{d}\omega \,, \tag{S16}$$

$$\mathcal{S}^{\mathrm{or}}_{\mathrm{ww}}(r,r') = \frac{\mathrm{R}}{\Omega^2} \int g^{\mathrm{or}}_{\mathrm{iw}}(\omega|r) g^{\mathrm{or}}_{\mathrm{iw}}(\omega'|r') \ln g^{\mathrm{or}}_{\mathrm{ww}}(\omega,\omega'|r,r') \,\mathrm{d}\omega\mathrm{d}\omega' \,. \tag{S17}$$

The total orientational entropy then follows from integrating the kernel $\mathcal{S}^{\mathrm{or}}$ over the respective spatial coordinates,

$$S^{\mathrm{or}}_{\mathrm{iw}} = -2\pi n_0 \int g^{\mathrm{r}}_{\mathrm{iw}}(r) \mathcal{S}^{\mathrm{or}}_{\mathrm{iw}}(r) r^2 \,\mathrm{d}r \,, \tag{S18}$$

$$S^{\mathrm{or}}_{\mathrm{ww}} = -8\pi^2 n_0^2 \int g^{\mathrm{r}}_{\mathrm{iw}}(r) g^{\mathrm{r}}_{\mathrm{iw}}(r') g^{\mathrm{r}}_{\mathrm{ww}}(r,r') \mathcal{S}^{\mathrm{or}}_{\mathrm{ww}}(r,r') r^2 r'^2 \,\mathrm{d}r\mathrm{d}r' \,. \tag{S19}$$

Due to the systematic underestimation of the water-water orientational entropy observed at finite sampling of the pair correlations as discussed in (*86*), the extrapolation method presented therein is used to obtain a reliable estimate for the water-water correlations at infinite sampling. Consequently, the quantity

$$\sigma(r,r';N_{\mathrm{conf}}) \equiv g^{\mathrm{r}}_{\mathrm{iw}}(r;N_{\mathrm{conf}})\, g^{\mathrm{r}}_{\mathrm{iw}}(r';N_{\mathrm{conf}})\, g^{\mathrm{r}}_{\mathrm{ww}}(r,r';N_{\mathrm{conf}})\, \mathcal{S}^{\mathrm{or}}_{\mathrm{ww}}(r,r';N_{\mathrm{conf}}) \tag{S20}$$

is extrapolated as a function of the number of sampled configurations $N_{\mathrm{conf}}$ from the inverse power-law

$$\sigma(r,r';N_{\mathrm{conf}}) = \sigma^*(r,r') + \frac{A(r,r')}{N_{\mathrm{conf}}^{\alpha(r,r')}} \,, \tag{S21}$$

where $\sigma^*(r,r')$, $A(r,r')$ and $\alpha(r,r')$ are $(r,r')$-dependent parameters that were determined through least-squares fitting of the MD data. Here, the quantity $\sigma^*(r,r')$ represents the (negative) local orientational entropy of ion solvation at infinite sampling. The fit was carried out over 10 sets of data corresponding to values $N_{\mathrm{conf}} = 1, 2, \ldots, 10 \times N^*_{\mathrm{conf}}$, with individual sample sizes $N^*_{\mathrm{conf}}$ ranging between $30 \times 10^6$ and $40 \times 10^6$ for all the different ion types in the bulk and at the interface. Furthermore, to get rid of (tiny) long-range correlations that artificially blow up the integral in Eq. (S19) due to the Jacobian $r^2 r'^2$, for all water-water pairs where neither of the molecules is located within the first hydration shell of the ion, $\sigma^*(r,r') = 0$ is applied. For the calculation of the ion-water entropy, the histogram underlying the ion-water pair correlation function $g_{\mathrm{iw}}(r,\omega)$, was generated from MD trajectories using bin sizes of $\Delta r = 0.2\,\mathrm{\AA}$ and $\Delta\omega = 10^\circ$ for the respective spatial and orientational coordinates. To enhance the statistics for the water-water entropy, a larger spatial bin size $\Delta r = 0.4\,\mathrm{\AA}$ was utilized in the computation of $g_{\mathrm{iww}}(r,r',\theta,\theta')$. The obtained values for bulk and interface entropy of $Cs^+$, $Li^+$, $Ca^{2+}$ electrolytes are summarized in Table S2.

For better visualization of the ion's impact on water structure, we further analyzed water-water PCFs in the Kirkwood Superposition Approximations (KSA), which assumes that the inhomogeneous PCF is

equivalent to the unperturbed bulk water PCF (*33*), that depends only on the mutual distance $r$ and the relative orientation $\omega_{\text{rel}}$ between two water molecules,

$$g_{\text{ww}}^{\text{inh}}(\mathbf{r}, \mathbf{r}', \omega, \omega') = g_{\text{ww}}^{0}(r, \omega_{\text{rel}}) \, . \tag{S22}$$

The five relative angles $\omega_{\text{rel}}$ are defined in Fig. S6b. Similar to the inhomogeneous case, Eqs. (S16) and (S17), the translational and orientational contributions can be separated; see (*32, 86*) for details. The 2D orientational distribution functions (ODFs) for pure bulk and interface water with dissolved $Cs^+$, $Li^+$ and $Ca^{2+}$ in Fig. 2 and Fig. S7 are then obtained from

$$g(\omega_i, \omega_j | r) = \frac{\int g(\omega_{\text{rel}}|r) \, \text{d}\omega_{k \neq i,j}}{\int \text{d}\omega_{k \neq i,j}} \, . \tag{S23}$$

Here, $\omega_i$ and $\omega_j$ denote the angles describing the relative orientation of two molecules ($i \neq j$), and $\text{d}\omega_{k \neq i,j}$ represents the integration over all angles other than $\omega_i$ or $\omega_j$. The 2D distributions represent the projection of the full ODF onto two dimensions (*32*).

## Supplementary Text 3. Three-body-angle distribution

The distributions of three-body-angles $\gamma$ are sampled from MD trajectories following the definition given in Fig. S8a and using a bin size of 4.5°. The obtained three-body-angle distributions for different ion types in the bulk and at the interface are shown in Fig. S8b. In the bulk, the three-body-structure is very similar for $Cs^+$, $Li^+$ and $Ca^{2+}$, with a broadened peak around 109°, corresponding to perfect tetrahedral alignment of water molecules. A smaller second peak emerges at around 50°, which primarily corresponds to the appearance of a 5th neighbor within the first hydration shell that sits interstitial to the predominately tetrahedral arrangement (*34*). At the interface, the three-body-angle distributions become drastically different compared to the bulk with the maxima of the distribution shifting towards smaller $\gamma$. However, this effect is more pronounced for the strongly hydrated $Li^+$ and $Ca^{2+}$ ions where a significant new maximum of the distribution appears around 60°. Such a peak is characteristic of Lennard-Jones liquids with no polarity (*34*), indicating that water dipole-dipole interactions are effectively dominated by the strong ion-dipole interaction that ultimately allows for a denser packing due to the increased appearance of interstitial water molecules compared to the bulk. The effect is weaker for $Cs^+$, for which water maintains more of its tetrahedral structure and the distribution shows a plateau between 50° and 109°.

To quantify the effect of the different ion types on the water structure, we assume that three-body-angles between 75° and 138° correspond to a tetrahedral configuration as proposed in (*87*). In consonance with the similar distribution functions in the bulk, for all ion types approximately 67% of water molecules are in a tetrahedral configuration. For adsorbed $Cs^+$, still 62 % of water molecules at the interface are in a tetrahedral configuration, whereas for $Li^+$ and $Ca^{2+}$ the number is reduced to 58 % and 59 %, respectively.

## Supplementary Text 4. Influence of sample absorbance and interference on sum frequency generation spectroscopy

In our experimental configuration for conventional and phase-resolved sum frequency generation (SFG) spectroscopy the laser beams centred at 640 nm (broadband, SFG light), 800 nm (narrowband) and 3100 nm (broadband) have to pass through the mica substrate before reaching the mica-water interface. Mica absorbs light at all used wavelengths, although at different magnitudes. Consequently, we had to use thin substrates, ranging between ca. 130-180 $\mu$m, for the measurements to limit the absorption of the used beams. Fig. S10 depicts ultraviolet-visible and infrared transmission spectra of selected mica substrates with different thicknesses. In spite of the steep decrease of the light transmission below 600 nm seen in Fig. S10a, there is negligible absorption of the 800 nm and SFG light. On the contrary, in the region of the broadband infrared beam between 2800-3600 nm, shown in Fig. S10b, the transmission varies strongly between samples and further features peaks/dips, which likely explain the observed peak-like features of the mica-$D_2O$ interface displayed in Fig. 3a. For a discussion of the band assignment, we refer to the already existing literature (*88*). Besides, a fringe pattern is visible in Fig. S10b whenever the transmission is higher than ca. 0.3. These fringes originate from the interference of the initially transmitted infrared beam with beams undergoing multiple reflections within the mica substrate. Such interference effects were also observed for the detected SFG light. For substrates thinner than ca. 140 $\mu$m clear interference patterns were observed in conventional SFG spectra, as shown in Fig. S10c. For thicker substrates the interference pattern was not clearly visible, most likely due to the higher frequency of the fringes or less overlap of the interfering beams on the detector. In phase-resolved SFG spectroscopy the unwanted interference terms could be removed during the processing of the spectra.

## Supplementary Text 5. Superposition of the mica-air and mica-liquid signals in sum frequency generation spectroscopy

A challenge for measuring thin substrates is that the sum frequency signal of the top mica-air interface cannot be separated on the detector from the signal of the bottom mica-liquid interface. The measured response is thus potentially a combination of both, as sketched in Fig. S11a. Conventional sum frequency generation (SFG) spectroscopy indicated that the signal in the hydrogen bonded OH-stretch region (3000-3500 $cm^{-1}$) comes mostly from the mica-liquid interface, because of the strong intensity difference between the mica-$D_2O$ and mica-$H_2O$ spectra depicted in Fig. 3a. Above 3500 $cm^{-1}$ hydroxyls within and at the surface of the mica crystal were found in literature (*89*). However, the strong absorption (Fig. S10b) and superposition of mica-air and mica-liquid signals prevent a discussion of our measurements in that frequency region. Below 3000 $cm^{-1}$ we found CH-stretch vibrations, which are assumed to come from organic traces on the mica-air interface and not from the freshly cleaved mica surface in contact with the liquid. This assumption

is supported by a comparison of an intensity spectrum from conventional SFG spectroscopy with the magnitude squared of the phase-resolved spectrum, which is displayed in Fig. S11b. Both spectra feature the mica-water interface and should ideally overlap quantitatively. Yet, the CH-stretch vibrations (below 3000 $cm^{-1}$) and mica-hydroxyl groups (above 3500 $cm^{-1}$) have significantly higher intensities in the magnitude squared phase-resolved data when it is scaled to the hydrogen bonded OH-stretch region (3000-3500 $cm^{-1}$). This intensity mismatch is most likely caused by the strength of the local oscillator. The local oscillator light reflected from the top mica-air surface has roughly 10 times more power than the reflected local oscillator light from the bottom mica-water interface (sketched in Fig. S11a). The intensity in phase-resolved SFG spectroscopy is determined by the magnitude of the interference between the local oscillator and the sample signal, which is proportional to the respective power of both beams. The higher power of the local oscillator light reflected at the mica-air interface therefore boosted the vibrational peaks from this interface (CH-stretch vibrations and mica-hydroxyls), while the signal from the mica-water interface (bonded OH-stretch region) was markedly less enhanced.

The different scaling of the mica-air and mica-water interface contributions in the phase-resolved data complicates a detailed analysis. Additionally, phase-resolved SFG spectroscopy requires that the phase between the local oscillator and quartz reference electromagnetic waves is the same as the phase between the local oscillator and the sample SFG signal (Fig. S11a, $\Delta_{top}$, $\Delta_{bottom}$, $\Delta_{mix}$). A difference of the phase due to experimental limitations has to be corrected in the postprocessing of the spectra. However, a uniform correction is not possible in the case of thin mica samples since the three phases $\Delta_{top}$, $\Delta_{bottom}$ and $\Delta_{mix}$ are all different. A derivation for this phase difference is given at the end of this section for interested readers. As the focus of this study is on the bonded OH-stretch region, we corrected the spectra so that the imaginary part of the second order nonlinear susceptibility ($Im(\chi^{(2)})$) of the mica-$D_2O$ spectrum is as flat as possible between 3000-3500 $cm^{-1}$, as depicted in Fig. S9a-c. Still, the spectral shape and individual peaks should be interpreted with utmost care and we will limit the following discussion only on qualitative trends. Similar to the conventional SFG spectra shown in Fig. 3a, the addition of $CsNO_3$, $LiNO_3$ and $CaCl_2$ causes the $Im(\chi^{(2)})$ to decrease in the bonded OH-stretch region. The intensity reduction follows again the trend: $LiNO_3$ < $CaCl_2$ < $CsNO_3$, where the spectrum of mica in contact with $CsNO_3$ matches with the spectrum of the mica-$D_2O$ interface. Furthermore, it can be seen that the intensity/sign after adding $LiNO_3$ and $CaCl_2$ lies between the mica-$D_2O$ and mica-$H_2O$ spectra, which implies that there is no flip of the net water dipole orientation upon salt addition.

In the following, we will show in more detail how the mica substrate affects the phase differences between the local oscillator SFG light and the SFG light from the mica interfaces. The calculations are similar to literature (*90*). As a simplification we base our model on the real part of the refractive index for unpolarised

light, which neglects the birefringence and light absorption of mica. The refractive index for the 800 nm ($n_{800nm}$=1.77) and SFG beam ($n_{640nm}$=1.46) were taken from literature (*91*), while the refractive index of the IR beam was calculated based on the spacing of the interference pattern in the transmission infrared spectra, shown in Fig. S10b, according to the following equation:

$$n_{mica} = \frac{1}{2d \cdot \left(\frac{1}{\lambda_2} - \frac{1}{\lambda_1}\right)} \tag{S24}$$

Where $n_{mica}$ is the real part of the refractive index, $d$ is the thickness of the mica substrate and $\lambda_1$ and $\lambda_2$ are the wavelengths of two adjacent maxima or minima in the interference pattern. As such, the refractive index of mica in the infrared was determined as $n_{3100nm}$=1.58±0.07 (average of 7 samples ± standard deviation). In accordance with literature (*90*), we can write the phase Δ of each beam as

$$\Delta(\lambda) = \frac{2 \cdot \pi \cdot l \cdot n}{\lambda} \tag{S25}$$

with $l$ as the path length through the medium and $n$ as the real refractive index for the respective wavelength $\lambda$. For the phase-resolved measurements it is of interest if there is a substantial difference between the phase of the local oscillator light reflected from the top surface with the mica-air interface SFG light (Fig. S11a, $\Delta_{top}$) and the phase of the mica-liquid SFG light either interfering with the bottom ($\Delta_{bottom}$) or top reflection ($\Delta_{mix}$) of the local oscillator. The three individual phases can be defined at the point of the mica-air interface as follows:

$$\Delta_{top} = 0 \tag{S26}$$

$$\Delta_{bottom} = \Delta_{800nm} + \Delta_{3100nm} + \Delta_{640nm} - 2 \cdot \Delta_{640nm} \tag{S27}$$

$$\Delta_{mix} = \Delta_{800nm} + \Delta_{3100nm} + \Delta_{640nm} \tag{S28}$$

As a simplification, we set $\Delta_{top}$ arbitrarily to 0, as we are only interested in relative differences. Consequently, we can define two phase differences:

$$\Delta_{bottom-top} = \Delta_{bottom} - \Delta_{top} \tag{S29}$$

$$\Delta_{mix-top} = \Delta_{mix} - \Delta_{top} \tag{S30}$$

These two phase differences are shown as a function of the mica sample thickness in Fig. S9d and S9e. The periodicity of the phase difference is 3.13$\mu$m for $\Delta_{bottom-top}$ and 0.17$\mu$m for $\Delta_{mix-top}$. This is unfortunately significantly too small to allow for a trustworthy absolute determination of the phase difference for specific mica samples depending on their thickness and thus limits the reliability of the performed phase correction in the processing of the phase-resolved SFG data.

## Supplementary Text 6. Interfacial electrostatic properties for SFG data interpretation

The interfacial electrostatic properties are examined using a simplified continuum model based on data sampled from the MD simulations. It is assumed that the system is homogeneous in the lateral directions, such that the electric potential only depends on the $z$-direction perpendicular to the mica surface, which is further calculated from Poisson's equation,

$$-\varepsilon_0\varepsilon_\perp \frac{\mathrm{d}^2\Phi(z)}{\mathrm{d}z^2} = \rho(z) - \frac{\mathrm{d}P(z)}{\mathrm{d}z} \,. \tag{S31}$$

Because the behavior of the perpendicular relative permeability $\varepsilon_\perp$ at solid–liquid interfaces under high salt concentrations remains insufficiently characterized (*92*), and experimental data for the mica–water interface at low salt concentrations indicate qualitatively similar profiles $\varepsilon_\perp(z)$ across different salts (*93*), we adopt the constant value of bulk water $\varepsilon_\perp \approx 78.4$ for all ion species. The ionic charge density $\rho(z)$ is computed from the cation/anion number density profiles $n_i(z)$ obtained from MD simulations, $\rho(z) = \mathrm{e}_0(\nu\, n_{\mathrm{cation}}(z) - n_{\mathrm{anion}}(z))$, where $\mathrm{e}_0$ and $\nu$ are the elementary charge and ion valency, respectively. The net water polarization density $P(z)$ with respect to the mica surface normal is approximated using

$$P(z) = \mu\, n_{\mathrm{w}}(z)\, \langle \cos\alpha \rangle(z) \,, \tag{S32}$$

where $\mu \approx 6.17 \times 10^{-30}\,\mathrm{C\,m}$ is the dipole moment of the applied SPCE water force field, $n_{\mathrm{w}}(z)$ is the MD sampled water density profile, $\alpha$ is the angle between the water dipole vector and the mica surface normal vector, and $\langle\cdot\rangle$ denotes ensemble average.

The computed profiles of $\rho(z)$, $P(z)$ and $\Phi(z)$ are shown in Fig. S4. To check the robustness of the presented method, we further calculated the surface charge density,

$$\sigma = -\varepsilon_0\varepsilon_\perp \left.\frac{\mathrm{d}\Phi}{\mathrm{d}z}\right|_{z=0} \approx -0.34\,\mathrm{C\,m^{-2}} \tag{S33}$$

for all ion types, which is close to the applied force field parameters of the MD simulations.

Including local electric field effects, the square-root of the total SFG intensity, $\sqrt{I_{\mathrm{MD}}} = |E_{\mathrm{SFG}}|$, can be approximated as

$$E_{\mathrm{SFG}} \propto \chi^{(2)} + \chi^{(3)} \cdot \Phi(z=0), \tag{S34}$$

where $\chi^{(2)}$ and $\chi^{(3)}$ are the second- and third-order contributions, and $\Phi(z=0)$ is the surface potential. In the here applied formalism, $\chi^{(2)}$ contains the signal contribution by oriented water molecules, while $\chi^{(3)}$, equal for all ion types, represents the potential-induced polarization of water molecules, not accessible to the MD simulations. Interference terms, often included, are negligible at 500 mM salt concentrations (*37–39*). For estimating the expected experimental SFG intensity, we now integrate the water polarization density $P(z)$ (figure S4b) from the surface to the bulk. The integral $\int P(z)dz$, relating to $\chi^{(2)}$, is numerically quoted

in the top corners of Figure 3b and shows a large positive value for $Cs^+$ and a negative and smaller value for $Li^+$ and $Ca^{2+}$, respectively. The positive (negative) sign correlates with water on average oriented with the hydrogen pointing away from (towards) the mica surface, as discussed in the main section. The larger absolute $\chi^{(2)}$ value of the integral for $Cs^+$ compared to the other two ions is not directly in line with the experimental SFG observations showing the smallest intensities ($\sqrt{I_{\mathrm{EXP}}}$, corners of Figure 3a) for $Cs^+$.

However, at charged interfaces the electric field induced by the surface charge can also polarize the water molecules in the EDL, not resulting in a change of the orientation (*38, 45*). This effect, associated with the $\chi^{(3)}$ contribution, is not directly included in MD simulations, but can be estimated from MD-based surface potentials (see figure S4c for electric potential profiles).

For the surface potential $\Phi(z = 0)$, we obtain from MD simulations values for $Cs^+$, $Li^+$, and $Ca^{2+}$ of 7.15, −89.8 and −82.8 mV, respectively. Further, using the experimental observation of zero SFG signal for $Cs^+$ and the integral of the polarization as $\chi^{(2)}$ (i.e. 0.95 for $Cs^+$) in Equation (S34), the $\chi^{(3)}$ value and then the expected intensities $\sqrt{I_{MD}}$ can be determined. Based on $\sqrt{I_{MD}}$ we obtain a ratio of 1.1 for the SFG intensity of $Li^+$ and $Ca^{2+}$. In comparison, the experimental $Li^+/Ca^{2+}$ ratio was obtained by integrating the 3000–3500 cm$^{-1}$ signal, subtracting the $D_2O$ interface response, and normalizing to the water interface. Taking the square root of the integrated intensity (quoted in figure 3a) yields an experimental ratio of ~ 1.7 (average of two datasets), slightly larger than the MD-based estimation. However, the trend of smaller signal for $Ca^{2+}$ *vs* $Li^+$ is comparable. Please note that the determination of the potential at the surface is not trivial; literature even suggests extrapolation of it from the bulk potential (*45*).

## Supplementary Text 7. Ion-water and water-water PCFs and entropy results

Fig. 2a presents the calculated ion-water RDFs $g^{\mathrm{r}}_{\mathrm{iw}}(r)$ for $Cs^+$, $Li^+$ and $Ca^{2+}$ in the bulk and at the interface. The corresponding values for the hydration shell radius $r_{\mathrm{shell}}$, coordination number $N_{\mathrm{c}}$ (nr. of water molecules in the first hydration shell), and volumetric density $n_{\mathrm{vol}}$ of hydration are summarized in table S1. The latter quantity is defined as $n_{\mathrm{vol}} = 3\,N_{\mathrm{c}}/(4\pi r^3_{\mathrm{shell}})$. The hydration shell radius $r_{\mathrm{shell}}$ is determined by the first minimum in $g^{\mathrm{r}}_{\mathrm{iw}}(r)$ and increases with ion size: $Li^+ < Ca^{2+} < Cs^+$. Consequently, $N_{\mathrm{c}}$ is obtained by spherical integration of the RDF from 0 to $r_{\mathrm{shell}}$

$$N_{\mathrm{c}} = 4\pi\, n_0 \int_0^{r_{\mathrm{shell}}} g^{\mathrm{r}}_{\mathrm{iw}}(r)\, r^2 \mathrm{d}r\,, \tag{S35}$$

where $n_0$ is the bulk number density of water.

The magnitude of the first peak in the ion-water RDF $g^{\mathrm{r}}_{\mathrm{iw}}(r)$ in 2a indicates that the strength of ion hydration follows the order $Cs^+ < Li^+ < Ca^{2+}$, corresponding to (negative) hydration energies in the order $Ca^{2+} < Li^+ < Cs^+$. Despite the weaker ion-water interaction in the bulk, $Cs^+$, due to its size, carries a relatively large, low density hydration shell that binds the largest number of water molecules. This changes during the adsorption process: Although $r_{\mathrm{shell}}$ stays constant, the coordination number changes for all ion types. The

larger peaks in $g^{\mathrm{r}}_{\mathrm{iw}}(r)$ for adsorbed $Li^+$ and $Ca^{2+}$ are due to increased packing of water molecules within the first hydration shell, while the peak for $Cs^+$ decreases, indicating effective dehydration of the adsorbed ion. The latter stems from comparable magnitudes of $Cs^+$-water and water-water interaction strengths obtained from the corresponding RDFs in Fig. 2a and 2b. Thereby, water molecules can be expelled more easily from the hydration shell of $Cs^+$. This preference for water-water interactions effectively dehydrates the interface, consistent with the observed signal loss in the SFG spectra in Fig. 3a. In contrast, $Li^+$ and $Ca^{2+}$ exhibit an increase in hydration number and density, with ion-water interactions dominating over water-water interactions, ultimately packing more water at the interface.

Hydration strength also directly affects the orientational distribution functions of the first hydration shell calculated from

$$g^{\mathrm{or}}_{\mathrm{iw}}(\omega_i) = \frac{1}{\Omega} \int_0^{\pi} \int_0^{r_{\mathrm{shell}}} g_{\mathrm{iw}}(r, \omega) r^2 \mathrm{d}r \mathrm{d}\omega_j \; . \tag{S36}$$

Here, $\omega_i$ represents the ion-water angle of interest, such that the integration is performed over the remaining angle $\omega_j$. The obtained distribution functions $g^{\mathrm{or}}_{\mathrm{iw}}(\theta)$ and $g^{\mathrm{or}}_{\mathrm{iw}}(\chi)$ are presented in Fig. S12a and S12b, respectively. In general, both orientational distribution functions (ODFs) are more uniform and relatively unstructured for the weakly hydrated $Cs^+$, whereas for $Li^+$ and $Ca^{2+}$ the ODFs display distinct peaks, reflecting localized or strongly favored configurations. This indicates that increased ion-dipole interaction yields stronger ion-water orientational correlations. In Fig. S12a, for $Cs^+$ the water dipole vectors are only weakly aligned with the ion's electric field, which results in a much broader and an order of magnitude lower peak of $g^{\mathrm{or}}_{\mathrm{iw}}(\theta)$ for small $\theta$ compared to $Li^+$ and $Ca^{2+}$ with distinct peaks close to $\theta \approx 0$. Upon adsorption these maxima diminish for all ion types. Additionally, for $Cs^+$, the distribution function changes qualitatively with a new maximum appearing around 60°. The $g^{\mathrm{or}}_{\mathrm{iw}}(\chi)$ in Fig. S12b are qualitatively similar between $Cs^+$, $Li^+$ and $Ca^{2+}$, only differing in the flatness and offset to the bulk distribution. For adsorbed $Cs^+$ the offset is slightly smaller compared to bulk, with no significant change to the flatness of the distribution. Adsorbed $Li^+$ in contrast shows more structuring in the ODF, with smaller populations around $\chi \approx 90°$ but larger populations at the distribution's edges. The latter is also observed for $Ca^{2+}$.

The ion-water translational and orientational entropy for $Cs^+$, $Li^+$ and $Ca^{2+}$, obtained from the above distribution functions in the bulk and at the interface, are summarized in Table S2. Generally, in the bulk and at the interface, ion-water entropy decreases with stronger hydration interaction, which indicates that the energetics of ion hydration directly influences the ordering of water molecules in the vicinity of the solute. This picture is in line with results for bulk systems published by Shi *et. al* (*25*). Upon adsorption, $Li^+$ and $Ca^{2+}$ show a decrease in the ion-water translational order due to the attraction of additional water molecules into their first hydration shell and corresponding increase of the first peak in $g^{\mathrm{r}}_{\mathrm{iw}}(r)$. Due to its dehydration, $Cs^+$ ultimately shows no entropic penalty and the translational entropy is larger at the interface compared to bulk. At the interface, orientational entropy values are rather similar to the bulk for all ion types. Looking at the kernel function $\mathcal{S}^{\mathrm{or}}_{\mathrm{iw}}(r)$ from Eq. (S16), shown in Fig. S12c we find that orientational

entropy contributions in the first hydration shell are actually enhanced for adsorbed ions. The slight decrease of the overall orientational entropy for $Cs^+$ stems from the increased long range interactions that become apparent when plotting the integrand of Eq. (S18) in Fig. S12d. Here, for $Li^+$ and $Ca^{2+}$, the increase in $S^{or}_{iw}(r)$ is compensated by increased hydration, *i.e.* more water molecules contribute to the orientational correlations compared to bulk. The tendency of $Li^+$ and $Ca^{2+}$ to attract more water molecules into their first hydration shell upon adsorption, ultimately determines the ion-water contribution to their corresponding entropic penalty.

The water-water entropies in Table S2 reveal a similar picture compared to the ion-water correlations – a large hydration energy yields a small entropy value, corresponding to highly ordered water structures – following the trend $Cs^+ > Li^+ > Ca^{2+}$. The latter pays a high entropic penalty for both translational and orientational correlations, whereas $Li^+$ only significantly alters the orientational part of the total entropy change upon adsorption. $Cs^+$ shows a comparatively small delta in both the translational and orientational entropy.

The extrapolated function $\sigma^*(r, r')$, Eq. (S21) used to evaluate the orientational water-water entropy for $Cs^+$, $Li^+$ and $Ca^{2+}$ in the bulk and at the interface using Eq. (S19) is depicted in Fig. S13. Consistent with the ion-water results, water-water orientational correlations follow $Cs^+ < Li^+ < Ca^{2+}$, corresponding to smaller entropy values and therefore stronger orientational water-water ordering induced by the more strongly hydrated ions. Almost no qualitative and quantitative change between bulk and interface $\sigma^*(r, r')$ is observed for the weakly hydrated $Cs^+$, in line with the small delta of the orientational entropy in Table S2. For the $Li^+$ ion, a quantiative change of $\sigma^*(r, r')$ is observed for $r = r' \approx r_{\text{shell}}$, indicating that water-water orientational correlations are affected for molecule pairs that both sit in the first hydration shell, accompanied with a significant entropy delta upon adsorption. For $Ca^{2+}$, the distribution of bulk and interface differ both qualitatively and quantitatively, showing both a severe increase of correlations in the first hydration shell and, additionally, for pairs where one of the molecules sits outside of the first hydration shell. This ultimately yields the largest entropic penalty for $Ca^{2+}$ adsorption.

Fig. S7 shows the orientational water structure for pure bulk and interfacial water with adsorbed $Cs^+$, $Li^+$ and $Ca^{2+}$ ions based on the other five 2D ODFs of hydration shell waters obtained from Eq. (S23), in addition to the ODF in Fig. 2d. We consider the interface up to 4 Å away from the surface. The reduced overall intensity for the interface ODFs stems from the smaller number of water pairs that are formed at the interface. Results for pure bulk water are similar to results published in the original publication (*32*), although minor differences appear due the different water model that has been used in this study. Qualitative comparison of $g^{or}_{ww}(\theta_1, \theta_2)$ in Fig. 2d reveals that $Li^+$ and $Ca^{2+}$ induce new correlations in $g^{or}_{ww}(\theta_1, \theta_2)$ as a consequence of the water dipoles' alignment with the ion's electric field. For $Cs^+$ only minor qualitative changes appear in $g^{or}_{ww}(\theta_1, \theta_2)$, indicating that the tetrahedral water structure is maintained at the interface. The bulk and interfacial ODFs in Fig. S7a, S7b and S7c remain qualitatively similar to the bulk for all

adsorbed ions. Intensity variations stem from the varying number of water pairs that are formed at the interface. Due to the strong electric field of $Ca^{2+}$, new interface configurations are also found in $g^{\text{or}}_{\text{ww}}(\phi, \chi_2)$ and $g^{\text{or}}_{\text{ww}}(\theta_1, \phi)$, shown in Fig. S7d and S7e, respectively. Here, no change is observed for $Cs^+$ and $Li^+$, underlining the ion-specific effect on water structure that follows the Hofmeister series $Cs^+ < Li^+ < Ca^{2+}$.

## Supplementary Text 8. Pairwise summation of electrostatic interactions at the interface

To quantify the counterbalancing electrostatic interactions at the interface, we employ a classical pairwise summation model, between nearest neighbor interactions. In this framework, the free energy contributions are expressed as Coulombic interaction energies between charged species (ion–ion and ion–surface) and ion–dipole interactions with interfacial water molecules. Each contribution depends explicitly on the respective separation distance and, in the case of ion–dipole coupling, on the relative orientation of the dipole. The total stabilizing interaction energy is hence written as the sum of these terms:

$$\sum W_i = W_{\text{ion-ion}}(r_{\text{ion–ion}}) + W_{\text{ion-surf}}(r_{\text{ion–surface}}) + W_{\text{ion-dipole}}(r_{\text{ion-dipole}}, \theta) \tag{S37}$$

Where the individual terms are calculated as follows:

$$W_{\text{ion-ion}}(r_{\text{ion-ion}}) = +\frac{(z_{ion}e)^2}{4\pi\varepsilon_0\varepsilon\, r_{\text{ion-ion}}}. \tag{S38}$$

$$W_{\text{ion-surf}}(r_{\text{ion-surf}}) = -\frac{z_{\text{ion}}z_{\text{surface}}e^2}{4\pi\varepsilon_0\varepsilon\, r_{\text{ion–surface}}}. \tag{S39}$$

$$W_{\text{ion-dipole}}(r_{\text{ion-dipole}}, \theta) = -\delta n\frac{z_{\text{ion}}e\,\mu\cos\theta}{4\pi\varepsilon_0\varepsilon\, r^2_{\text{ion-dipole}}}. \tag{S40}$$

All distances are approximated as time-averaged values obtained from molecular dynamics (MD) simulations at 500 mM concentration, as well as AFM micrographs. Specifically, the ion-ion distance corresponds to the average nearest-neighbor separation between ions, derived from the Delaunay triangulations in figure S3. The ion-surface distance is the average separation between ions and the negative charge centers at the surface, and the ion-dipole interaction is evaluated from the change in interfacial water density ($\delta n$) estimated from the MD simulations (see Table S1).

As can be seen in the equations, this leaves a single free parameter, namely the dielectric permittivity $\varepsilon$, in Eq. S37. Under equilibrium conditions, where $\Delta G = 0$ and thus $\Delta H \approx \sum W_i = T\Delta S$, we can therefore fit $\varepsilon$ for each ion. One should note, we are omitting the relatively weak attractive van der Waals interactions, as well as dipole-dipole interactions in this first order approximation, as they are largely overpowered by the accounted Coulomb contributions. Including these will only have a minor effect on the fit of $\varepsilon$ and will not change

the main conclusion. Yet these contributions may become very important in other systems, such as metal surfaces, where for instance van der Waals interactions are generally about an order of magnitude larger. Dipole-dipole enthalpic contributions are weak in general, in comparison to other enthalpic contributions.

# Supplementary Figures

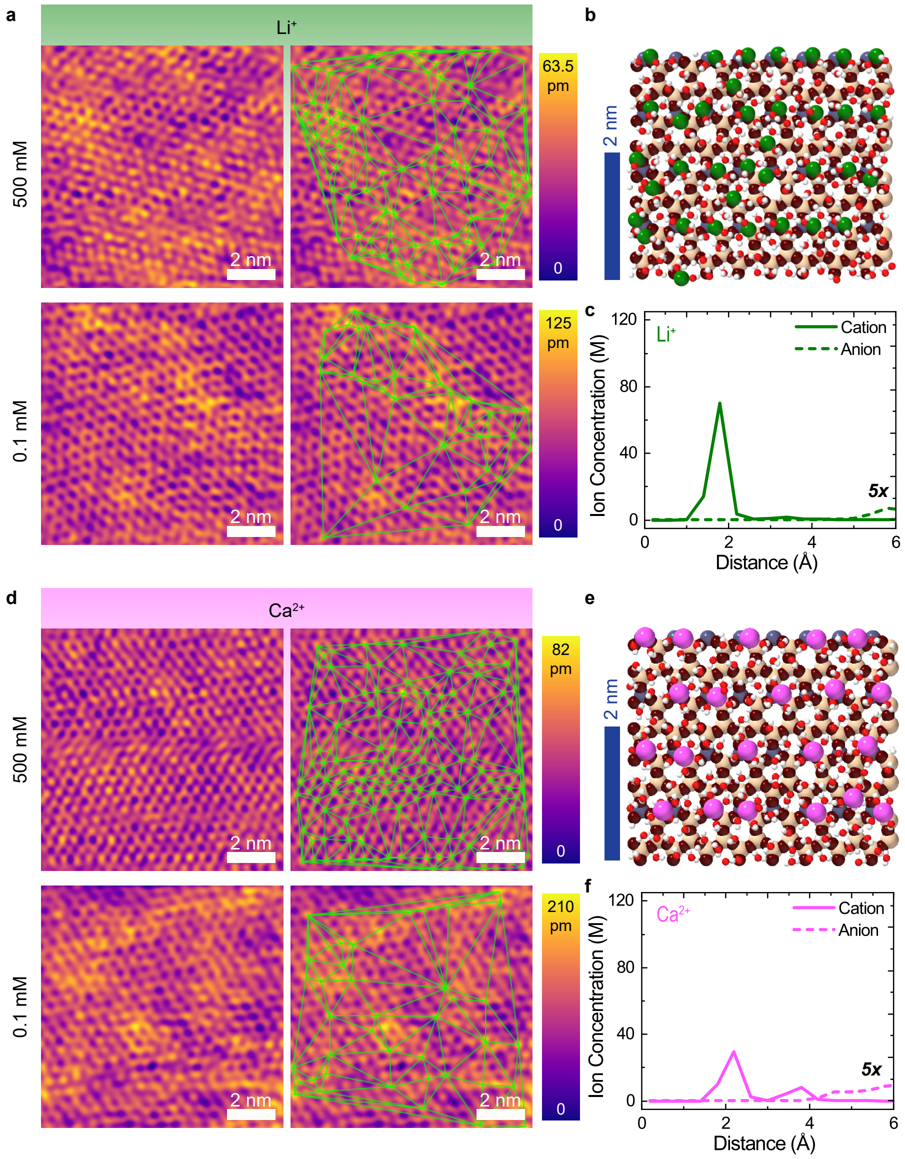


**Figure S1**: **Visualization and quantification of $Li^{+}$ and $Ca^{2+}$ adsorption on muscovite mica.** (**a**) and (**d**) show AFM images and corresponding Delaunay traingulation patterns (green lines) for mica exposed to 500 mM and 0.1 mM $Li^{+}$ and $Ca^{2+}$, respectively. (**b**) and (**e**) show molecular dynamics snapshot of mica exposed to a solution of $Li^{+}$ and $Ca^{2+}$ at 500 mM, respectively. (**c**) and (**f**) display the interfacial ion concentration profiles *vs* distance from the surface for $Li^{+}$ and $Ca^{2+}$ at 500 mM, respectively.

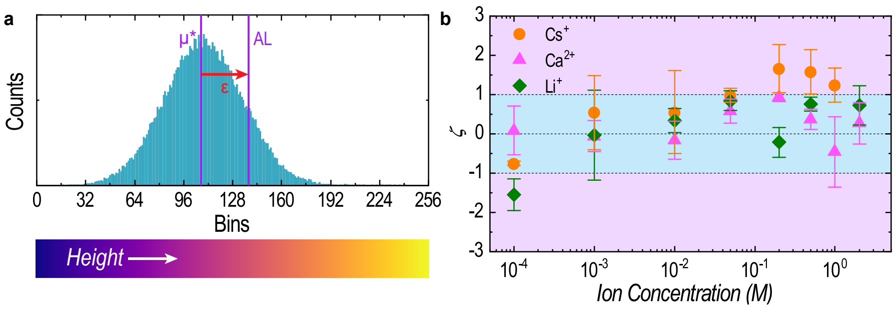


**Figure S2**: **Adsorption layer definition from AFM topographies.** (**a**) Pixels distribution histogram of a typical AFM image of highly resolved mica. From the histogram, $\mu^*$ indicates the mode of the distribution, while AL indicates the threshold corresponding to the adsoprtion layer. $\varepsilon$ is the displacement from the modal plane that defines the adsoprtion layer as a multiple integer of the standard deviation (see Supplementary Text). (**b**) Skewed distribution parameter $\zeta$ (see equation S3) as a function of the cation concentration in solution. Areas in lilac ( -1< $\zeta$ and $\zeta \geq 1$) indicate pronouncedly skewed distributions, while the area in blue (-1 $\leq \zeta$ <1) indicates *quasi*-normal distribution.

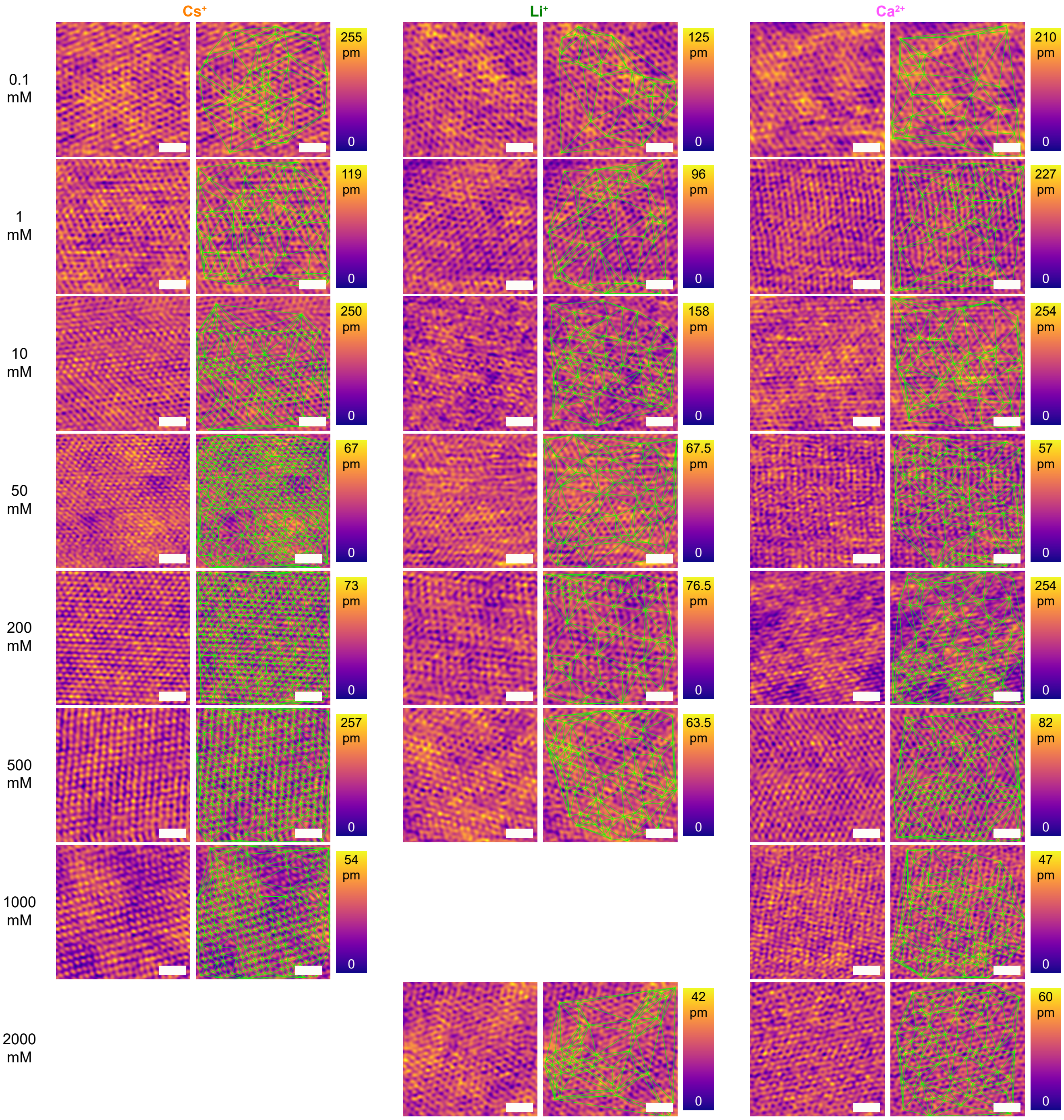


**Figure S3**: **Adsorption quantification of $Cs^+$, $Li^+$ and $Ca^{2+}$ on mica at different concentrations.** AFM topographies of ions ($Cs^+$, $Li^+$ and $Ca^{2+}$) adsorbed on mica at different ionic strengths. Images in the left column represent the high-resolution micrographs, whereas the images in the right column display the corresponding Delaunay triangulation patterns (green lines) calculated, as described in Supplementary Text. The scale bar in all images represents 2 nm.

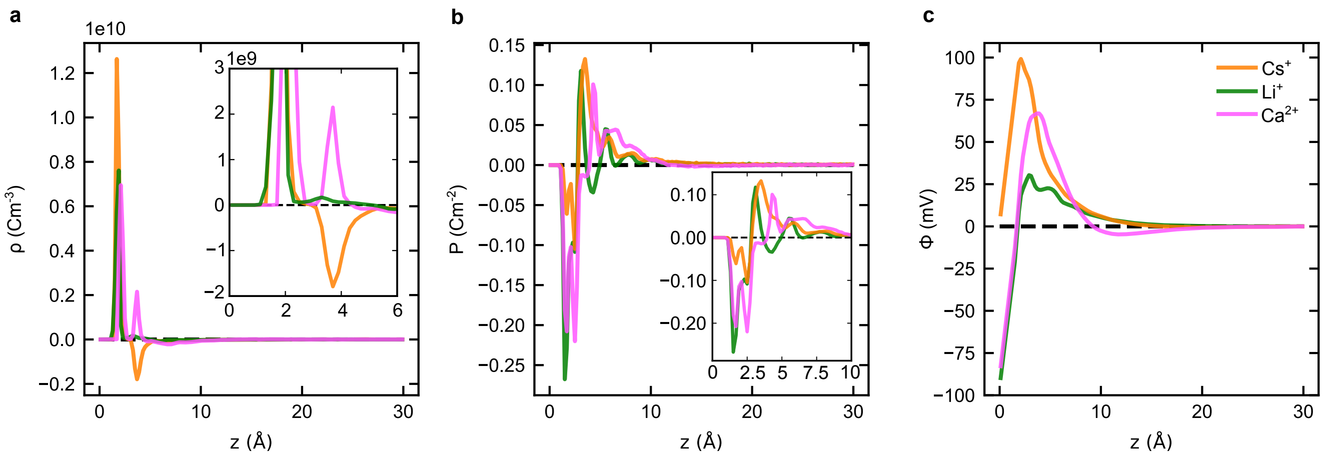


**Figure S4**: **Interfacial electrostatic properties.** (**a**) Ion net charge density profile for different salt solutions. The inset gives a closer view on the second layer. Due to the almost perfect screening by $Li^+$, the net charge is close to zero after the first ion layer. Contrastingly, the overscreening by $Cs^+$ is compensated with negative ion charges in the second layer. Since $Ca^{2+}$ does not entirely screen the negative mica charge, a second layer of positive ion charges emerges. (**b**) Polarization density computed from Eq. (S32) for different salt solutions. The inset shows a magnified plot of the interface region. (**c**) Electric potentials calculated from Poisson's equation (S31). The negatively charged mica surface is located at $z = 0$.

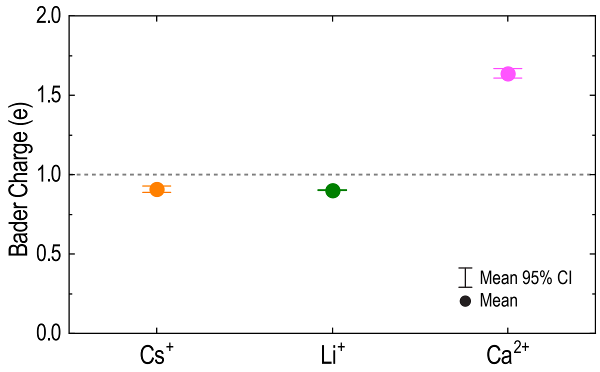


**Figure S5**: **Bader charge analysis.** The electronic and ionic minimization of the DFT algorithm leads to positive charges in the Cs, Li, and Ca atoms upon interaction with the water and mica slab. Cs and Li lose almost a full electron compared to their neutral state, justifying the treatment of these species as $Cs^+$ and $Li^+$, while Ca gains almost a +2 charge.

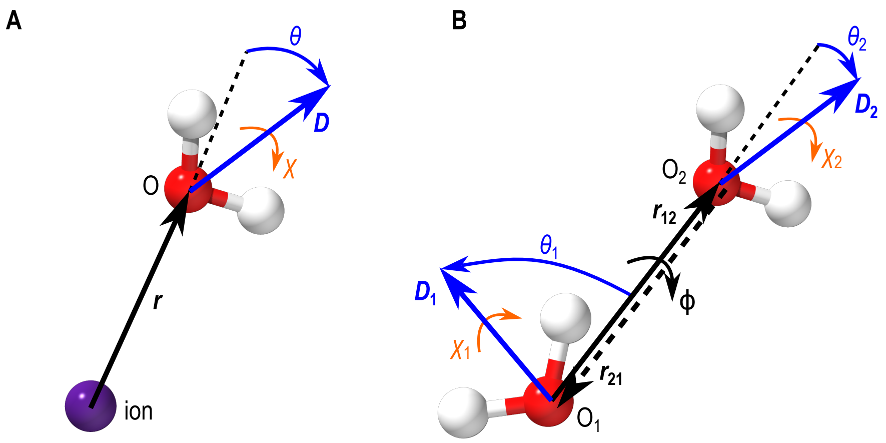


**Figure S6**: **Pair correlation functions.** Definition of (**a**) the ion-water angles $\omega = \theta, \chi$ (*33*) and (**b**) the relative water-water angles $\omega_{\mathrm{rel}} = \theta_1, \theta_2, \phi, \chi_1, \chi_2$ in the Kirkwood Superposition Approximation (KSA) (*32*).

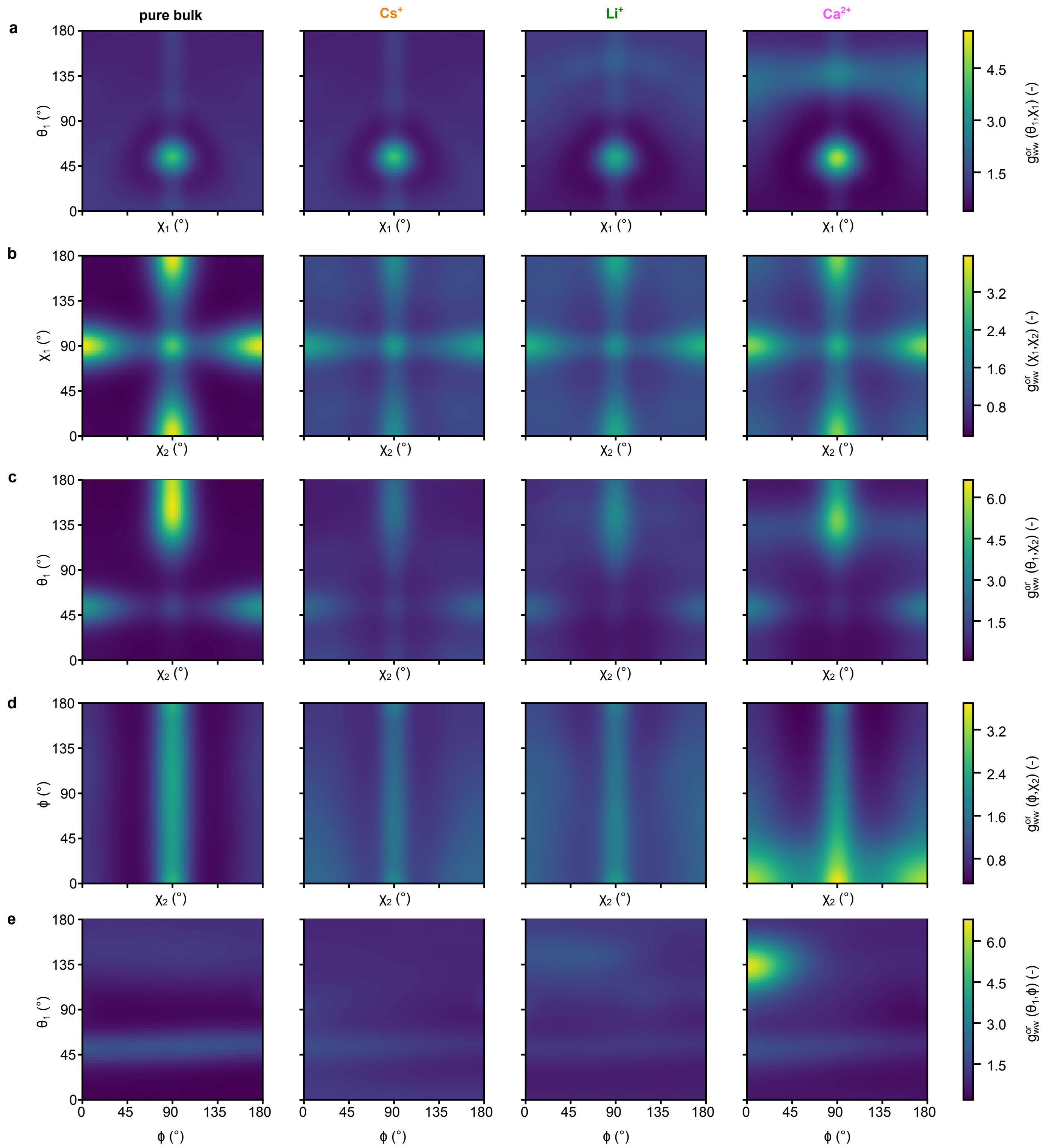


**Figure S7**: **Orientational structure of water.** Comparison of water-water ODFs in pure bulk water and at the interface for adsorbed $Cs^+$, $Li^+$ and $Ca^{2+}$: (**a**) $g^{\mathrm{or}}_{\mathrm{ww}}(\theta_1, \chi 1)$, (**b**) $g^{\mathrm{or}}_{\mathrm{ww}}(\chi_1, \chi_2)$, (**c**) $g^{\mathrm{or}}_{\mathrm{ww}}(\theta_1, \chi_2)$, (**d**) $g^{\mathrm{or}}_{\mathrm{ww}}(\phi, \chi_1)$, and (**e**) $g^{\mathrm{or}}_{\mathrm{ww}}(\theta_1, \phi)$. We consider the interface is up to 4 Å away from the surface and applied the Kirkwood Superposition Approximation (KSA) (*33*), with the corresponding correlation functions defined in Fig. S6(**b**). Since fewer water pairs are formed at the interface, an overall intensity loss is observed for all ions.

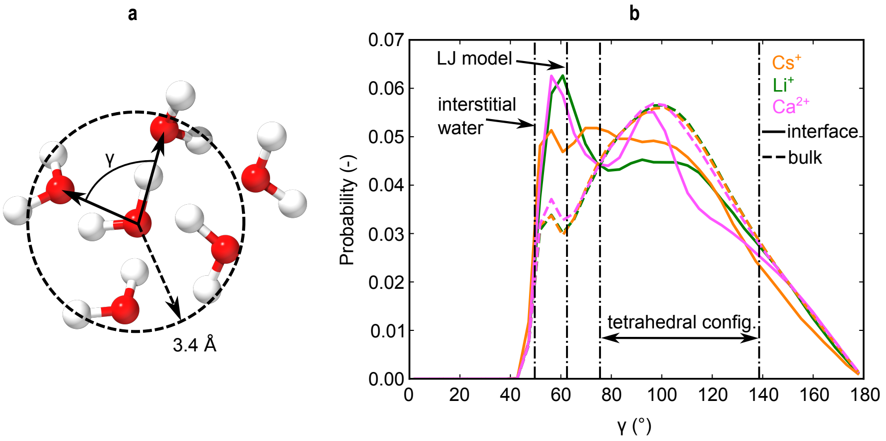


**Figure S8**: **Three-body water structure.** (**a**) Definition of the three-body-angle $\gamma$ between two vectors connecting a central water oxygen atom to oxygen atoms of two neighboring water molecules. For the computation of the three-body-angle distribution, all angles formed by unique pairs of water molecules within the 3.4 Å cutoff are considered. (**b**) Obtained three-body-angle distributions for different ion types in the bulk and at the interface. The tetrahedral configuration window delineates the regime $\gamma \in [75°, 138°]$, which we take as bounds for the tetrahedral configuration of water molecules as applied in (*87*). The vertical dash-dotted lines at 50° and 63° correspond to configurations of interstitial water and the distribution maximum of a Lennard-Jones water model from (*34*), respectively.

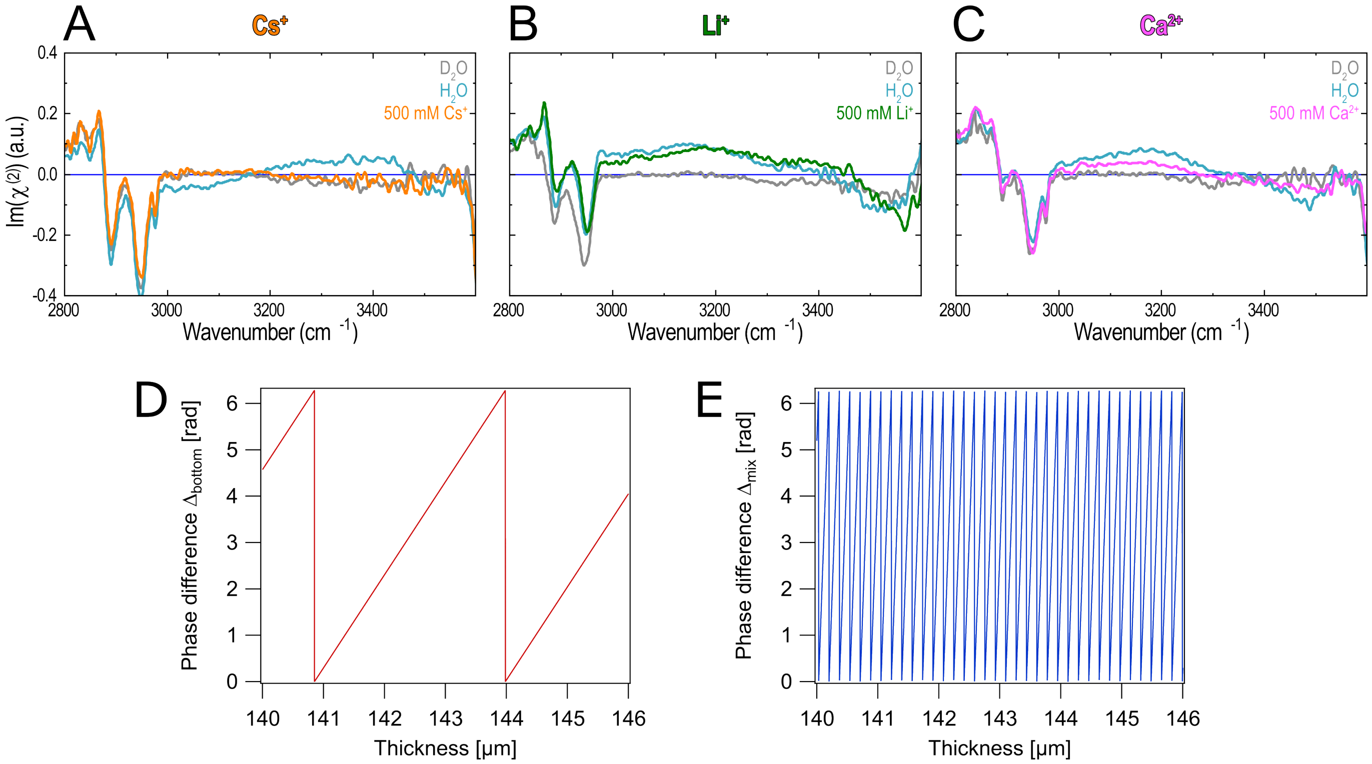


**Figure S9**: **Phase-resolved SFG results.** Phase-resolved SFG spectra of freshly cleaved mica in contact with first $D_2O$, then $H_2O$ and subsequently a 500 mM aqueous solution of (**a**) $CsNO_3$, (**b**) $LiNO_3$ and (**c**) $CaCl_2$. Calculations of the phase-difference between (**d**) $\Delta_{bottom}$ and (**e**) $\Delta_{mix}$, as defined in Equations S29 and S30.

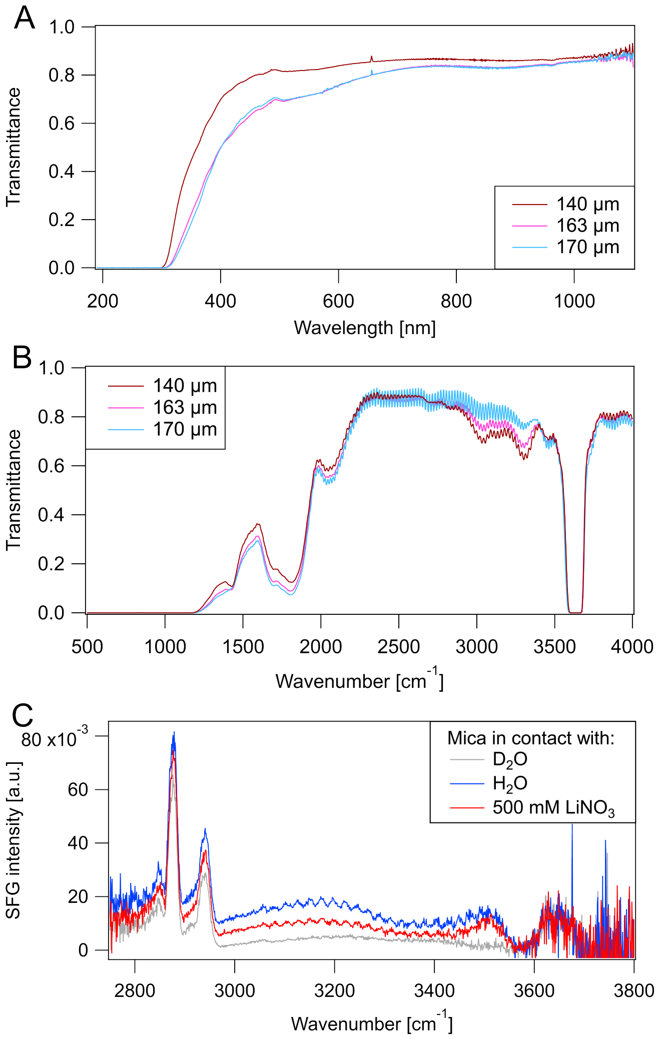


**Figure S10**: **Mica thickness effects.** Transmission spectra of mica substrates with selected thickness for (**a**) the ultraviolet-visible range and (**b**) the infrared region. (**c**) SFG spectra of mica in contact with $D_2O$, $H_2O$ and 500 mM $LiNO_3$. Around 3200 $cm^{-1}$ interference effects in the form of fringes from multiple reflections of the SFG light are visible.

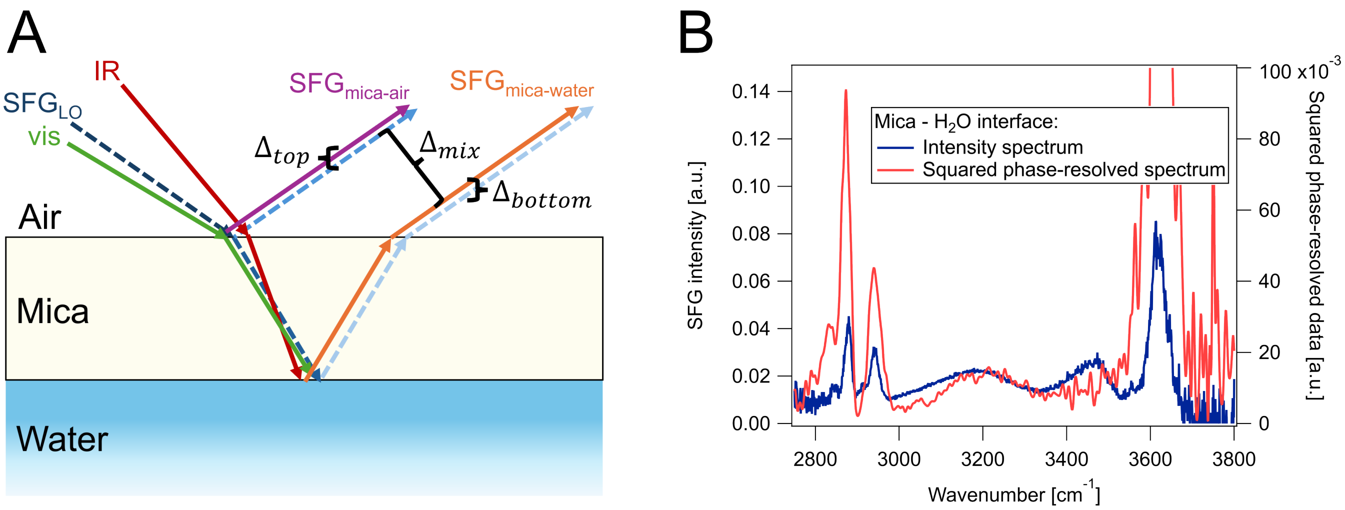


**Figure S11**: **Influence of the mica-air and mica-water interfaces on the SFG signal.** (**a**) Sketch of the centre of the laser beams used in SFG spectroscopy. The vis (800 nm) and IR (broadband infrared) beams overlap due to their estimated spot size diameter of around 100 µm at the mica-air and mica-water interface and produce SFG light at both interfaces. In phase-resolved experiments an additional SFG beam produced in the local oscillator generator (SFG$_{LO}$) overlaps with the SFG light produced at the mica substrate interfaces. The SFG$_{LO}$ reflected from the bottom mica-water interface is about 10 times weaker than the reflection at the mica-air interface. Additionally, the phase differences of the SFG$_{LO}$ light with the mica-air SFG signal ($\Delta_{top}$), the mica-water SFG signal ($\Delta_{bottom}$) and the mica-water signal with the top reflection of the SFG$_{LO}$ ($\Delta_{mix}$) are sketched. Multiple reflections inside the mica substrate are omitted for simplicity. (**b**) Comparison of a conventional intensity SFG spectrum and a magnitude squared phase-resolved spectrum.

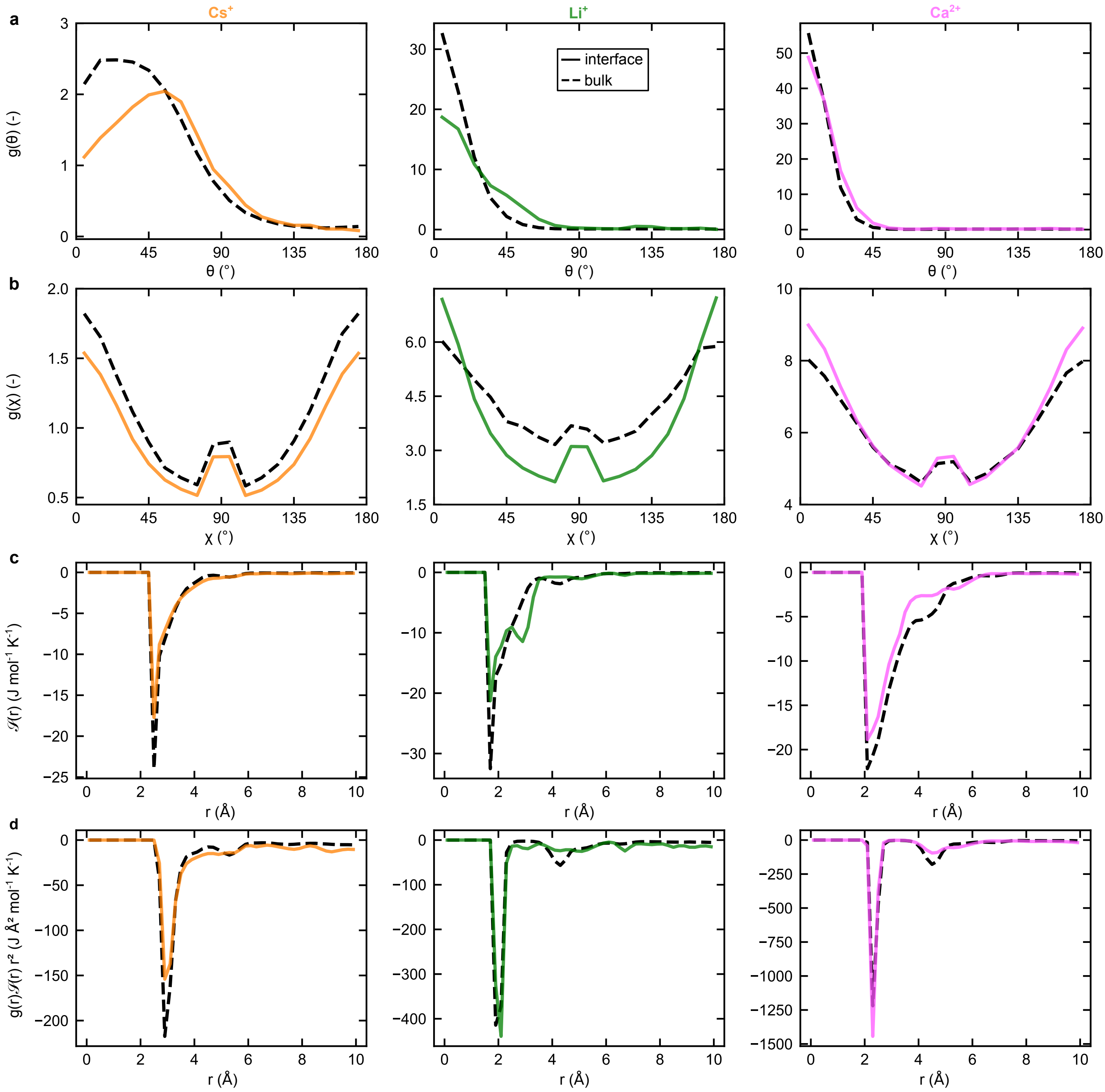


**Figure S12**: **Ion-water orientational distribution functions and entropy.** Comparison of ion-water orientational distribution functions (ODF), defined in Fig. S6(**a**), for $Cs^+$, $Li^+$ and $Ca^{2+}$: (**a**) $g^{\mathrm{or}}_{\mathrm{iw}}(\theta)$ and (**b**) $g^{\mathrm{or}}_{\mathrm{iw}}(\chi)$. ODFs are shown for water molecules sitting in the first hydration shell of the ion $r \leq r_{\mathrm{shell}}$. (**c**) Kernel functions $\mathcal{S}^{\mathrm{or}}_{\mathrm{iw}}$, Eq. (S16) for the calculation of the orientational ion-water entropy and (**d**) integrand for the calculation of the orientational ion-water entropy in Eq. (S18) for $Cs^+$, $Li^+$ and $Ca^{2+}$ in the bulk (full line) and at the interface. Continuous lines indicate the interface, while dashed lines indicate the bulk.

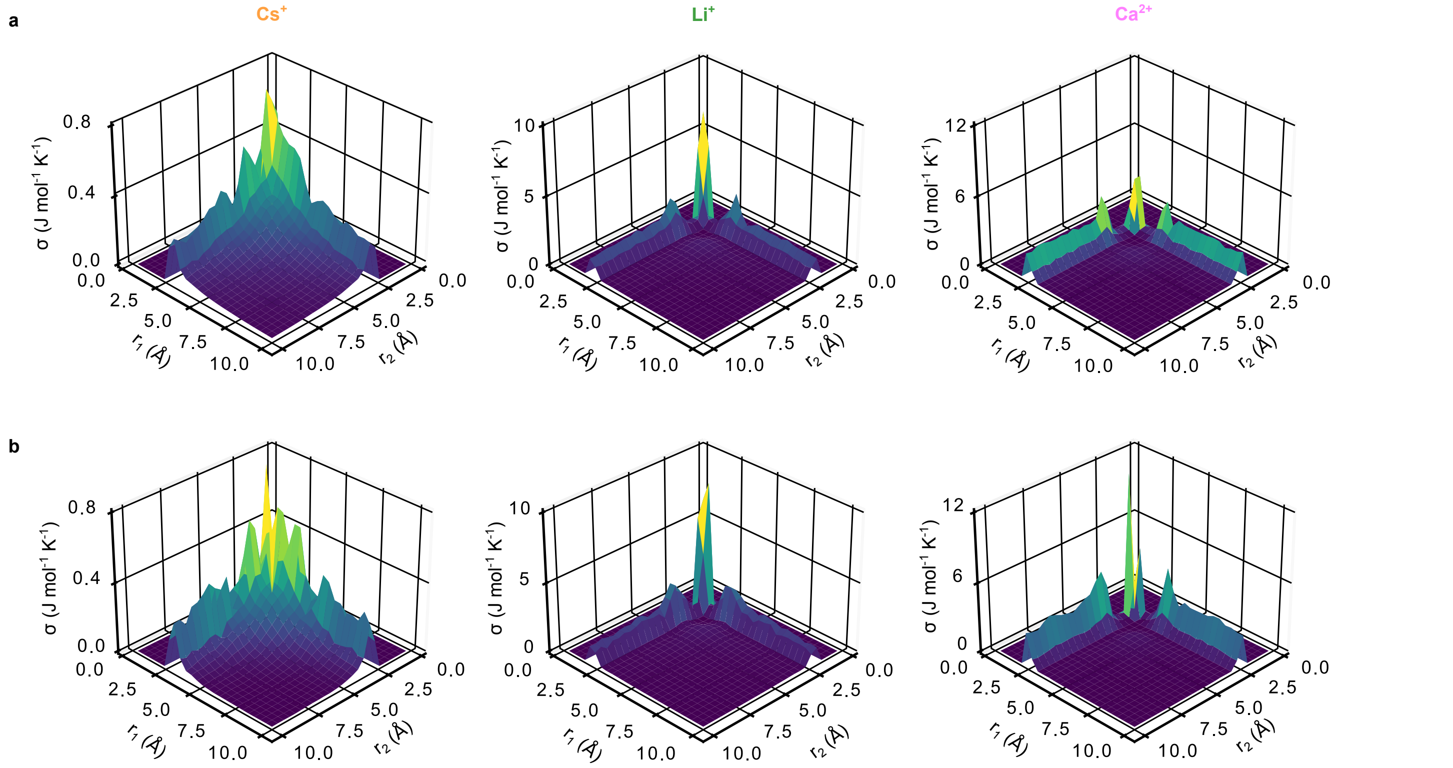


**Figure S13**: **Orientational entropy contributions from water-water pair correlations.** Comparison of the (negative) local orientational entropy of ion solvation $\sigma^*(r_1, r_2)$, Eq. (S21), used to calculate the orientational entropy contributions of $Cs^+$, $Li^+$ and $Ca^{2+}$ in **(a)** the bulk and **(b)** at the interface. Here, $(r, r')$ denotes the positions of the water molecules. Note that the vertical axes use different scalings depending on the ion type.

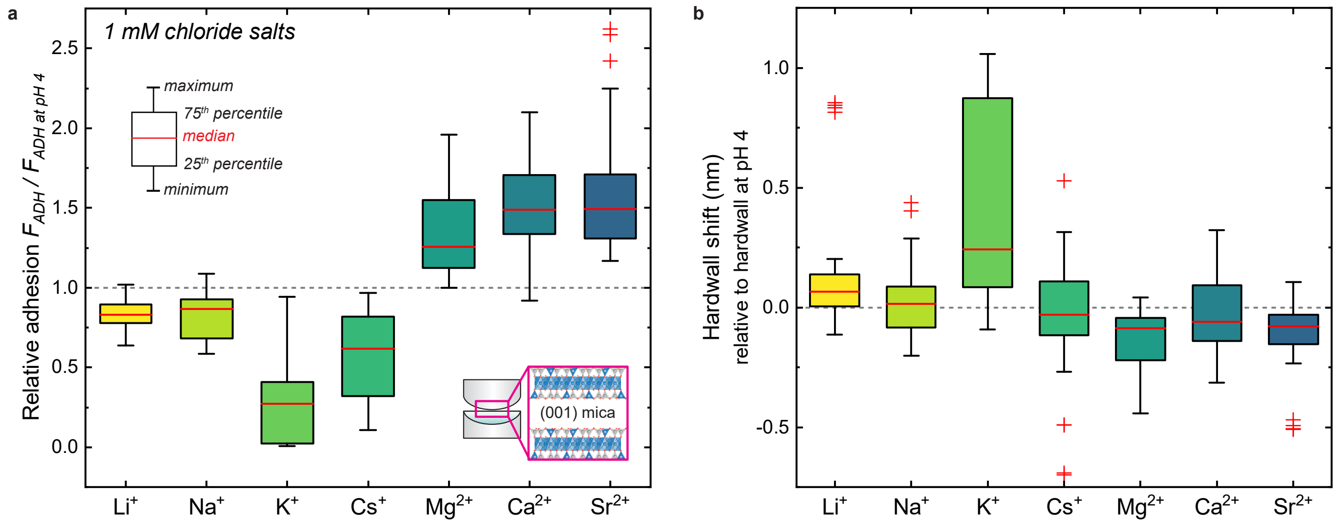


**Figure S14**: **Ion-dependent adsorption on mica regulates adhesive properties**. Surface force apparatus measurements of cation-dependent adhesion between two (001) muscovite mica surfaces. (**a**) Box and whiskers plot shows pull-off adhesion force for each 1 mM chloride salt with combined measurements from five independent experiments, normalized by the average adhesion measured in pH 4 HCl solution as described in the Methods section. (**b**) Box and whiskers plot comparing hardwall contact position shift for each 1 mM chloride salt in the same experiments as shown in panel **(a)**, reported as a difference with respect to the average hardwall position measured in pH 4 HCl solution as described in the Methods section. Red pluses in panels a and b are outliers.

# Supplementary Tables

**Table S1**: **Ion-water and water-water hydration characteristics.** Comparison of (first) hydration shell radius $r_{shell}$, coordination number $N_c$, and volumetric number (hydration) density $n_{vol}$ for ion-water and water-water interactions of $Cs^+$, $Li^+$, and $Ca^{2+}$ in the bulk and at the interface.

| | | ion-water | | | water-water | | |
|---|---|---|---|---|---|---|---|
| | | $Cs^+$ | $Li^+$ | $Ca^{2+}$ | $Cs^+$ | $Li^+$ | $Ca^{2+}$ |
| **bulk** | $r_{shell}$ (Å) | 3.9 | 2.7 | 3.1 | 3.3 | 3.3 | 3.3 |
| | $N_c$ (-) | 7.69 | 4.22 | 7.2 | 3.91 | 3.94 | 3.99 |
| | $n_{vol}$ (Å$^{-3}$) | 0.0309 | 0.0512 | 0.0577 | 0.0260 | 0.0262 | 0.0265 |
| **interface** | $r_{shell}$ (Å) | 3.9 | 2.7 | 3.1 | 4.7 | 4.3 | 3.7 |
| | $N_c$ (-) | 7.28 | 5.48 | 8.85 | 16.3 | 13.7 | 9.58 |
| | $n_{vol}$ (Å$^{-3}$) | 0.0293 | 0.0665 | 0.0709 | 0.0375 | 0.0411 | 0.0452 |

**Table S2**: **$Cs^+$ exhibits the lowest entropic penalty upon adsorption.** Orientational, translational and total entropy of ion-water and water-water interactions for the bulk, the interface and the difference Δ upon adsorption. Bulk and interface values are with respect to pure water at 298 K.

| ($J\ mol^{-1}\ K^{-1}$) | | ion-water | | | water-water | | |
|---|---|---|---|---|---|---|---|
| | | **$Cs^+$** | **$Li^+$** | **$Ca^{2+}$** | **$Cs^+$** | **$Li^+$** | **$Ca^{2+}$** |
| **$S_{tr}$** | bulk | -38.5 | -66.9 | -130.6 | -37.4 | -175.6 | -439.7 |
| | interface | -35.7 | -77.0 | -165.8 | -54.4 | -187.3 | -555.5 |
| | **Δ** | **2.8** | **-10.1** | **-35.2** | **-17.0** | **-11.7** | **-115.8** |
| **$S_{or}$** | bulk | -62.0 | -102.6 | -225.4 | -274.1 | -397.5 | -738.2 |
| | interface | -65.3 | -113.9 | -226.5 | -284.4 | -454.8 | -884.2 |
| | **Δ** | **-3.3** | **-11.3** | **-1.1** | **-10.3** | **-57.3** | **-146.0** |
| **$S_{tot}$** | bulk | -100.5 | -169.5 | -356.0 | -311.5 | -573.1 | -1177.9 |
| | interface | -101.0 | -190.9 | -392.3 | -338.8 | -642.1 | -1439.7 |
| | **Δ** | **-0.5** | **-21.4** | **-36.3** | **-27.3** | **-69.0** | **-261.8** |

## REFERENCES


1. X. Mao, P. Brown, C. Červinka, G. Hazell, H. Li, Y. Ren, D. Chen, R. Atkin, J. Eastoe, I. Grillo, A. A. H. Padua, M. F. Costa Gomes, T. A. Hatton, Self-assembled nanostructures in ionic liquids facilitate charge storage at electrified interfaces. *Nat. Mater.* **18**, 1350–1357 (2019).

2. P. Li, Y. Jiang, Y. Hu, Y. Men, Y. Liu, W. Cai, S. Chen, Hydrogen bond network connectivity in the electric double layer dominates the kinetic pH effect in hydrogen electrocatalysis on Pt. *Nat. Catal.* **5**, 900–911 (2022).

3. N. Dhopatkar, A. P. Defante, A. Dhinojwala, Ice-like water supports hydration forces and eases sliding friction. *Sci. Adv.* **2**, e1600763 (2016).

4. W. Kunz, Specific ion effects in colloidal and biological systems. *Curr. Opin. Colloid Interface Sci.* **15**, 34–39 (2010).

5. P. Lo Nostro, B. W. Ninham, Hofmeister phenomena: An update on ion specificity in biology. *Chem. Rev.* **112**, 2286–2322 (2012).

6. Y. Wei, J. Yang, Y. Hu, Y. Kaspi, J. Nie, Quasi-invariance of tropical meridional surface temperature gradient in a wide range of climates. *Nat. Commun.***17**, 123 (2026).

7. X. Li, Y. Wei, X. Gao, Z. Zhang, Z. L. Wang, D. Wei, Harnessing triboiontronic Maxwell’s demon by triboelectric-induced polarization for efficient energy-information flow. *Joule* **9**, 101888 (2025).

8. I. R. Gould, D. Ege, J. E. Moser, S. Farid, Efficiencies of photoinduced electron-transfer reactions: Role of the Marcus inverted region in return electron transfer within geminate radical-ion pairs. *J. Am. Chem. Soc.* **112**, 4290–4301 (1990).

9. M. C. O. Monteiro, F. Dattila, B. Hagedoorn, R. García-Muelas, N. López, M. T. M. Koper, Absence of $CO_2$ electroreduction on copper, gold and silver electrodes without metal cations in solution. *Nat. Catal.* **4**, 654–662 (2021).

10. P. Li, Y.-L. Jiang, Y. Men, Y.-Z. Jiao, S. Chen, Kinetic cation effect in alkaline hydrogen electrocatalysis and double layer proton transfer. *Nat. Commun.* **16**, 1844 (2025).

11. U. Raviv, J. Klein, Fluidity of bound hydration layers. *Science* **297**, 1540–1543 (2002).

12. T. Han, W. Cao, Z. Xu, V. Adibnia, M. Olgiati, M. Valtiner, L. Ma, C. Zhang, M. Ma, J. Luo, X. Banquy, Hydration layer structure modulates superlubrication by trivalent $La^{3+}$ electrolytes. *Sci. Adv.* **9**, eadf3902 (2023).

13. P. Bilotto, A. M. Imre, D. Dworschak, L. L. E. Mears, M. Valtiner, Visualization of ion|surface binding and in situ evaluation of surface interaction free energies via competitive adsorption isotherms. *ACS Phys. Chem. Au.* **1**, 45–53 (2021).

14. M. Lund, P. Jungwirth, C. E. Woodward, Ion specific protein assembly and hydrophobic surface forces. *Phys. Rev. Lett.* **100**, 258105 (2008).

15. J. Lyklema, Lyotropic sequences in colloid stability revisited. *Adv. Colloid Interface Sci.* **100-102**, 1–12 (2003).

16. K. Collins, Charge density-dependent strength of hydration and biological structure. *Biophys. J.* **72**, 65–76 (1997).

17. Y. Marcus, Effect of ions on the structure of water: Structure making and breaking. *Chem. Rev.* **109**, 1346–1370 (2009).

18. J. Mähler, I. Persson, A study of the hydration of the alkali metal ions in aqueous solution. *Inorg. Chem.* **51**, 425–438 (2011).

19. M. Ricci, P. Spijker, K. Voïtchovsky, Water-induced correlation between single ions imaged at the solid–liquid interface. *Nat. Commun.* **5**, 4400 (2014).

20. Y. Tian, Y. Song, Y. Xia, J. Hong, Y. Huang, R. Ma, S. You, D. Guan, D. Cao, M. Zhao, J. Chen, C. Song, K. Liu, L.-M. Xu, Y. Q. Gao, E.-G. Wang, Y. Jiang, Nanoscale one-dimensional close packing of interfacial alkali ions driven by water-mediated attraction. *Nat. Nanotechnol.* **19**, 479–484 (2024).

21. Y. Tian, J. Luo, Q. Tang, H. Zha, R. D. Priestley, W. Hu, B. Zuo, Intramolecular dynamic coupling slows surface relaxation of polymer glasses. *Nat. Commun.* **15**, 6082 (2024).

22. S. S. Lee, A. Koishi, I. C. Bourg, P. Fenter, Ion correlations drive charge overscreening and heterogeneous nucleation at solid–aqueous electrolyte interfaces. *Proc. Natl. Acad. Sci. U.S.A.* **118**, e2105154118 (2021).

23. K. J. Tielrooij, N. Garcia-Araez, M. Bonn, H. J. Bakker, Cooperativity in ion hydration *Science* **328**, 1006–1009 (2010).

24. Y. Chen, Z. Han, X. Li, K. Lu, Lowering coefficient of friction in Cu alloys with stable gradient nanostructures. *Sci. Adv.* **2**, e1601942 (2016).

25. R. Shi, A. J. Cooper, H. Tanaka, Impact of hierarchical water dipole orderings on the dynamics of aqueous salt solutions. *Nat. Commun.* **14**, 4616 (2023).

26. S.-H. Loh, S. P. Jarvis, Visualization of ion distribution at the mica−electrolyte interface. *Langmuir* **26**, 9176–9178 (2010).

27. S. McDonald, A. Elbourne, G. G. Warr, R. Atkin, Metal ion adsorption at the ionic liquid–mica interface. *Nanoscale* **8**, 906–914 (2016).

28. G. Franceschi, P. Kocán, A. Conti, S. Brandstetter, J. Balajka, I. Sokolović, M. Valtiner, F. Mittendorfer, M. Schmid, M. Setvín, U. Diebold, Resolving the intrinsic short-range ordering of $K^+$ ions on cleaved muscovite mica. *Nat. Commun.* **14**, 208 (2023).

29. C. Park, P. A. Fenter, N. C. Sturchio, K. L. Nagy, Thermodynamics, interfacial structure, and pH hysteresis of $Rb^+$ and $Sr^{2+}$ adsorption at the muscovite (001)−Solution interface. *Langmuir* **24**, 13993–14004 (2008).

30. J. Neumann, S. S. Lee, E. J. Zhao, P. Fenter, Direct experimental observations of ion distributions during overcharging at the muscovite-water interface by adsorption of $Rb^+$ and Halides ($Cl^-$, $Br^-$, $I^-$) at high salinity. *ChemPhysChem* **24**, e202300545 (2023).

31. S. S. Lee, C. Park, N. C. Sturchio, P. Fenter, Nonclassical behavior in competitive ion adsorption at a charged solid–water interface. *J. Phys. Chem. Lett.* **11**, 4029–4035 (2020).

32. T. Lazaridis, M. Karplus, Orientational correlations and entropy in liquid water. *J. Chem. Phys.* **105**, 4294–4316 (1996).

33. T. Lazaridis, Solvent reorganization energy and entropy in hydrophobic hydration. *J. Phys. Chem. B* **104**, 4964–4979 (2000).

34. J. I. Monroe, M. S. Shell, Decoding signatures of structure, bulk thermodynamics, and solvation in three-body angle distributions of rigid water models. *J. Chem. Phys.* **151**, 094501 (2019).

35. P. B. Miranda, L. Xu, Y. R. Shen, M. Salmeron, Icelike water monolayer adsorbed on mica at room temperature. *Phys. Rev. Lett.* **81**, 5876–5879 (1998).

36. S. Jin, Y. Liu, M. Deiseroth, J. Liu, E. H. G. Backus, H. Li, H. Xue, L. Zhao, X. C. Zeng, M. Bonn, J. Wang, Use of ion exchange to regulate the heterogeneous ice nucleation efficiency of mica. *J. Am. Chem. Soc.* **142**, 17956–17965 (2020).

37. E. C. Y. Yan, Y. Liu, K. B. Eisenthal, New method for determination of surface potential of microscopic particles by second harmonic generation. *J. Phys. Chem. B* **102**, 6331 (1998), 6336.

38. G. Gonella, C. Lütgebaucks, A. G. F. de Beer, S. Roke, Second harmonic and sum-frequency generation from aqueous interfaces is modulated by interference. *J. Phys. Chem. C* **120**, 9165–9173 (2016).

39. P. E. Ohno, H. B. Chang, A. P. Spencer, Y. Liu, M. D. Boamah, H.F. Wang, F. M. Geiger, Beyond the Gouy–Chapman model with heterodyne-detected second harmonic generation. *J. Phys. Chem. Lett.* **10**, 2328–2334 (2019).

40. J. Hunger, J. Schaefer, P. Ober, T. Seki, Y. Wang, L. Prädel, Y. Nagata, M. Bonn, D. J. Bonthuis, E. H. G. Backus, Nature of cations critically affects water at the negatively charged silica interface. *J. Am. Chem. Soc.* **144**, 19726 (2022).

41. T. Baimpos, B. R. Shrestha, S. Raman, M. Valtiner, Effect of interfacial ion structuring on range and magnitude of electric double layer, hydration, and adhesive interactions between mica surfaces in 0.05–3 M $Li^+$ and $Cs^+$ electrolyte solutions. *Langmuir* **30**, 4322–4332 (2014).

42. S. R. van Lin, K. K. Grotz, I. Siretanu, N. Schwierz, F. Mugele, Ion-specific and pH-dependent hydration of mica–electrolyte interfaces. *Langmuir* **35**, 5737–5745 (2019).

43. I. Siretanu, S. R. van Lin, F. Mugele, Ion adsorption and hydration forces: A comparison of crystalline mica vs. amorphous silica surfaces. *Faraday Discuss.* **246**, 274–295 (2023).

44. R. Goldberg, L. Chai, S. Perkin, N. Kampf, J. Klein, Breakdown of hydration repulsion between charged surfaces in aqueous $Cs^+$ solutions. *Phys. Chem. Chem. Phys.* **10**, 4939–4945 (2008).

45. T. Joutsuka, T. Hirano, M. Sprik, A. Morita, Effects of third-order susceptibility in sum frequency generation spectra: A molecular dynamics study in liquid water. *Phys. Chem. Chem. Phys.* **20**, 3040–3053 (2018).

46. A. Ben-Naim, *Molecular Theory of Solutions* (Oxford Univ. Press, 2006).

47. J. R. Errington, P. G. Debenedetti, Relationship between structural order and the anomalies of liquid water. *Nature* **409**, 318–321 (2001).

48. P. Kumar, S. V. Buldyrev, H. E. Stanley, A tetrahedral entropy for water. *Proc. Natl. Acad. Sci. U.S.A.* **106**, 22130–22134 (2009).

49. N. Giovambattista, P. G. Debenedetti, P. J. Rossky, Effect of surface polarity on water contact angle and interfacial hydration structure. *J. Phys. Chem. B* **111**, 9581–9587 (2007).

50. L. Sapir, D. Harries, Is the depletion force entropic? Molecular crowding beyond steric interactions. *Curr. Opin. Colloid Interface Sci.* **20**, 3–10 (2015).

51. Y. C. Kim, J. Mittal, Crowding induced entropy-enthalpy compensation in protein association equilibria. *Phys. Rev. Lett.* **110**, 208102 (2013).

52. X. Wang, S. Y. Lee, K. Miller, R. Welbourn, I. Stocker, S. Clarke, M. Casford, P. Gutfreund, M. W. A. Skoda, Cation bridging studied by specular neutron reflection. *Langmuir* **29**, 5520–5527 (2013).

53. I. C. Bourg, S. S. Lee, P. Fenter, C. Tournassat, Stern layer structure and energetics at mica–water interfaces. *J. Phys. Chem. C* **121**, 9402–9412 (2017).

54. S. S. Lee, P. Fenter, C. Park, N. C. Sturchio, K. L. Nagy, Hydrated cation speciation at the muscovite (001)-water interface. *Langmuir* **26**, 16647–16651 (2010).

55. S. S. Lee, M. Schmidt, N. Laanait, N. C. Sturchio, P. Fenter, Investigation of structure, adsorption free energy, and overcharging behavior of trivalent yttrium adsorbed at the muscovite (001)–water interface. *J. Phys. Chem. C* **117**, 23738–23749 (2013).

56. N. Govindarajan, A. T. Chu, C. Hahn, Y. Surendranath, The overlooked role of adsorption isotherms in electrocatalysis. *Nat. Catal.* **8**, 1254–1259 (2025).

57. C. Y. Son, Z.-G. Wang, Image-charge effects on ion adsorption near aqueous interfaces. *Proc. Natl. Acad. Sci. U.S.A.* **118**, e2020615118 (2021).

58. A. T. Celebi, A. Beskok, Molecular and continuum transport perspectives on electroosmotic slip flows. *J. Phys. Chem. C* **122**, 9699–9709 (2018).

59. M. Rashwan, B. Rehl, A. Sthoer, A. M. Darlington, M. S. Azam, H. Zeng, Q. Liu, E. Tyrode, J. M. Gibbs, Structure of the silica/divalent electrolyte interface: molecular insight into charge inversion with increasing pH. *J. Phys. Chem. C* **124**, 26973–26981 (2020).

60. H. Vanselous, P. B. Petersen, Extending the capabilities of heterodyne-detected sum-frequency generation spectroscopy: Probing any interface in any polarization combination. *J. Phys. Chem. C* **120**, 8175–8184 (2016).

61. S. Nihonyanagi, S. Yamaguchi, T. Tahara, Direct evidence for orientational flip-flop of water molecules at charged interfaces: A heterodyne-detected vibrational sum frequency generation study. *J. Chem. Phys.* **130**, 204704 (2009).

62. V. Wieser, P. Bilotto, U. Ramach, H. Yuan, K. Schwenzfeier, H.W. Cheng, M. Valtiner, Novelin situsensing surface forces apparatus for measuring gold versus gold, hydrophobic, and biophysical interactions. *J. Vac. Sci. Technol. A* **39**, 023201 (2021).

63. J. N. Israelachvili, N. A. Alcantar, N. Maeda, T. E. Mates, M. Ruths, Preparing contamination-free mica substrates for surface characterization, force measurements, and imaging. *Langmuir* **20**, 3616–3622 (2004).

64. J. Dziadkowiec, A. Royne, Nanoscale forces between basal mica surfaces in dicarboxylic acid solutions: Implications for clay aggregation in the presence of soluble organic acids. *Langmuir* **36**, 14978–14990 (2020).

65. R. M. Espinosa-Marzal, T. Drobek, T. Balmer, M. P. Heuberger, Hydrated-ion ordering in electrical double layers. *Phys. Chem. Chem. Phys.* **14**, 6085 (2012).

66. K. A. Schwenzfeier, A. Erbe, P. Bilotto, M. Lengauer, C. Merola, H.W. Cheng, L. L. E. Mears, M. Valtiner, Optimizing multiple beam interferometry in the surface forces apparatus: Novel optics, reflection mode modeling, metal layer thicknesses, birefringence, and rotation of anisotropic layers. *Rev. Sci. Instrum.* **90**, 043908 (2019).

67. S. M. Richardson, J. W. Richardson, Crystal structure of a pink muscovite from Archer's Post, Kenya: Implications for reverse pleochroism in dioctahedral micas *Am. Mineral.* **67**, 69–75 (1982).

68. S. Plimpton, Fast parallel algorithms for short-range molecular dynamics. *J. Comput. Phys.* **117**, 1–19 (1995).

69. R. T. Cygan, J.-J. Liang, A. G. Kalinichev, Molecular models of hydroxide, oxyhydroxide, and clay phases and the development of a general force field. *J. Phys. Chem. B* **108**, 1255–1266 (2004).

70. H. J. Berendsen, J.-R. Grigera, T. P. Straatsma, The missing term in effective pair potentials. *J. Phys. Chem.* **91**, 6269–6271 (1987).

71. I. S. Joung, T. E. Cheatham III, Determination of alkali and halide monovalent ion parameters for use in explicitly solvated biomolecular simulations. *J. Phys. Chem. B* **112**, 9020–9041 (2008).

72. S. Mamatkulov, M. Fyta, R. R. Netz, Force fields for divalent cations based on single-ion and ion-pair properties. *J. Chem. Phys.* **138**, 024505 (2013).

73. K. Kobayashi, Y. Liang, T. Sakka, T. Matsuoka, Molecular dynamics study of salt–solution interface: Solubility and surface charge of salt in water. *J. Chem. Phys.* **140**, 144705 (2014).

74. S. Miyamoto, P. A. Kollman, Settle: An analytical version of the SHAKE and RATTLE algorithm for rigid water models. *J. Comput. Chem.* **13**, 952–962 (1992).

75. I.-C. Yeh, M. L. Berkowitz, Ewald summation for systems with slab geometry. *J. Chem. Phys.* **111**, 3155–3162 (1999).

76. G. Kresse, J. Furthmüller, Efficiency of ab-initio total energy calculations for metals and semiconductors using a plane-wave basis set. *Comput. Mater. Sci.* **6**, 15–50 (1996).

77. G. Kresse, J. Furthmüller, Efficient iterative schemes forab initiototal-energy calculations using a plane-wave basis set. *Phys. Rev. B* **54**, 11169–11186 (1996).

78. P. E. Blöchl, Projector augmented-wave method. *Phys. Rev. B* **50**, 17953–17979 (1994).

79. G. Kresse, D. Joubert, From ultrasoft pseudopotentials to the projector augmented-wave method. *Phys. Rev. B* **59**, 1758–1775 (1999).

80. J. P. Perdew, K. Burke, M. Ernzerhof, Generalized gradient approximation made simple. *Phys. Rev. Lett.* **77**, 3865–3868 (1996).

81. S. Grimme, J. Antony, S. Ehrlich, H. Krieg, A consistent and accurateab initioparametrization of density functional dispersion correction (DFT-D) for the 94 elements H-Pu. *J. Chem. Phys.* **132**, 154104 (2010).

82. J. D. Pack, H. J. Monkhorst, "Special points for Brillouin-zone integrations"—A reply. *Phys. Rev. B* **16**, 1748–1749 (1977).

83. E. Sanville, S. D. Kenny, R. Smith, G. Henkelman, Improved grid-based algorithm for Bader charge allocation. *J. Comput. Chem.* **28**, 899–908 (2007).

84. W. Tang, E. Sanville, G. Henkelman, A grid-based Bader analysis algorithm without lattice bias. *J. Phys. Condens. Matter* **21**, 084204 (2009).

85. U. Ramach, J. Lee, F. Altmann, M. Schussek, M. Olgiati, J. Dziadkowiec, L. L. E. Mears, A. T. Celebi, D. W. Lee, M. Valtiner, Real-time visualisation of ion exchange

in molecularly confined spaces where electric double layers overlap. *Faraday Discuss.* **246**, 487–507 (2023).

86. E. Giuffrè, S. Prestipino, F. Saija, A. M. Saitta, P. V. Giaquinta, Entropy from correlations in TIP4P water. *J. Chem. Theory Comput.* **6**, 625–636 (2010).

87. A. Chaimovich, M. S. Shell, Tetrahedrality and structural order for hydrophobic interactions in a coarse-grained water model. *Phys. Rev. E* **89**, 022140 (2014).

88. M. Sayin, H. G. Von Reichenbach, Infrared spectra of muscovites as affected by chemical composition, heating and particle size. *Clay Miner.* **13**, 241–254 (1978).

89. A. Tuladhar, Z. A. Chase, M. D. Baer, B. A. Legg, J. Tao, S. Zhang, A. D. Winkelman, Z. Wang, C. J. Mundy, J. J. de Yoreo, H.F. Wang, Direct observation of the orientational anisotropy of buried hydroxyl groups inside muscovite mica. *J. Am. Chem. Soc.* **141**, 2135–2142 (2019).

90. A. G. Lambert, D. J. Neivandt, A. M. Briggs, E. W. Usadi, P. B. Davies, Interference effects in sum frequency spectra from monolayers on composite dielectric/metal substrates. *J. Phys. Chem. B* **106**, 5461–5469 (2002).

91. E. B. Singleton, C. T. Shirkey, Optical constants in the IR from thin film interference and reflectance: The reststrahlen region of muscovite mica. *Appl. Optics* **22**, 185 (1983).

92. D. J. Bonthuis, S. Gekle, R. R. Netz, Dielectric profile of interfacial water and its effect on double-layer capacitance. *Phys. Rev. Lett.* **107**, 166102 (2011).

93. O. Teschke, G. Ceotto, E. F. de Souza, Dielectric exchange force: A convenient technique for measuring the interfacial water relative permittivity profile. *Phys. Chem. Chem. Phys.* **3**, 3761–3768 (2001).
